# Electrostatic Discovery Atomic Force Microscopy

**DOI:** 10.1021/acsnano.1c06840

**Published:** 2021-11-22

**Authors:** Niko Oinonen, Chen Xu, Benjamin Alldritt, Filippo Federici Canova, Fedor Urtev, Shuning Cai, Ondřej Krejčí, Juho Kannala, Peter Liljeroth, Adam S. Foster

**Affiliations:** † Department of Applied Physics, Aalto University, 00076 Aalto, Helsinki, Finland; ‡ Nanolayers Research Computing Ltd, London N12 0HL, United Kingdom; § Department of Computer Science, Aalto University, 00076 Aalto, Helsinki, Finland; ∥ WPI Nano Life Science Institute (WPI-NanoLSI), Kanazawa University, Kakuma-machi, Kanazawa 920-1192, Japan

**Keywords:** atomic force microscopy, machine learning, tip functionalization, chemical identification, electrostatics

## Abstract

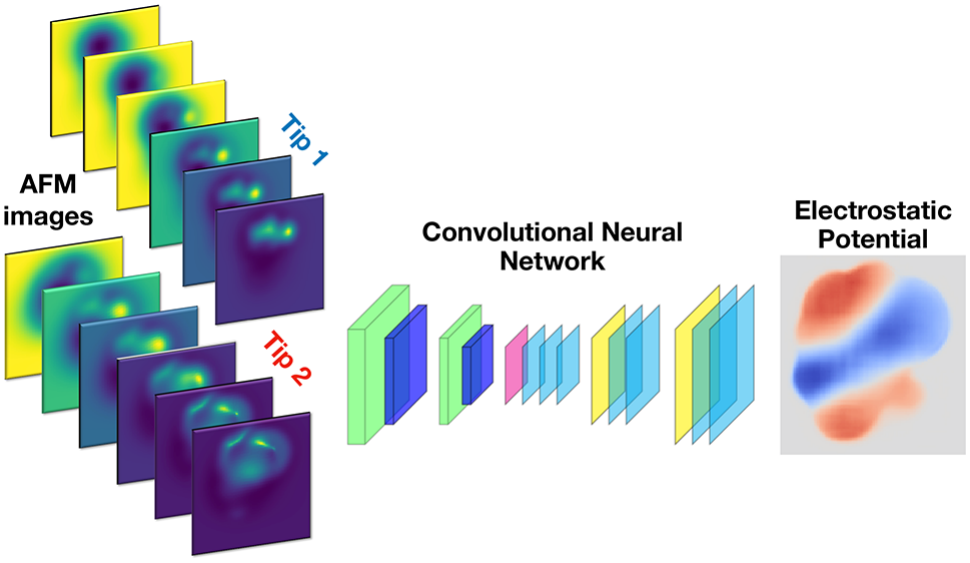

While offering high resolution atomic and electronic structure, scanning probe microscopy techniques have found greater challenges in providing reliable electrostatic characterization on the same scale. In this work, we offer electrostatic discovery atomic force microscopy, a machine learning based method which provides immediate maps of the electrostatic potential directly from atomic force microscopy images with functionalized tips. We apply this to characterize the electrostatic properties of a variety of molecular systems and compare directly to reference simulations, demonstrating good agreement. This approach offers reliable atomic scale electrostatic maps on any system with minimal computational overhead.

## Introduction

The electrostatic properties of molecules are dominant in a wide variety of processes and technologies, from catalysis and chemical reactions^1^ to molecular electronics^2^ and biological functions.^3^ In general, if we can understand the link between molecular function and electrostatics, it offers powerful tools to control and design functionality with nanoscale precision.^4^ On this scale, scanning probe microscopy (SPM) is the characterization technique of choice and scanning tunneling microscopy (STM) has become the engine of local electronic characterization for conducting systems,^5,6^ while AFM is a general tool for nanoscale imaging without material restrictions.^7,8^ In high-resolution studies, AFM has evolved from its origins^9^ into a breakthrough technique in studies of molecular systems.^10,11^ This has been driven by the use of functionalized tips, and AFM in ultra high vacuum (UHV) now offers a window into molecular structure on surfaces—aside from the detailed resolution of the results of molecular assembly, it is possible to study bond order, charge distributions, and the individual steps of on-surface chemical reactions.^11^ More recently, additions to the SPM family such as alternate-charging STM^12^ and single-electron transfer AFM^13^ have offered approaches to study charge behavior in molecular systems.

While all of these methods give indirect information on the electrostatic properties of the system being studied, significant efforts have been made to develop systematic techniques to directly characterize electrostatic properties. In particular, Kelvin probe microscopy (KPFM) was introduced^14,15^ to simultaneously explore the topography and local contact potential difference with atomic resolution. Despite success in characterizing the electrostatic properties of surfaces,^16−19^ and even proteins,^20^ the technique has limitations that prevent widespread adoption. Generally, it is experimentally challenging, requiring much longer measurement times than equivalent STM or AFM experiments and can be prone to tip-convolutions.^21^ More generally, the varying contributions to the signal mean it is very challenging to obtain quantitative measurements from KPFM.^22^ The step-change in molecular characterization offered by functionalized tips in AFM has also been harnessed for electrostatic analysis, with KPFM being applied with functionalized tips to provide a local potential maps of single molecules.^23,24^ However, the outstanding challenges of KPFM remain, or are even exaggerated: there is no rigorous KPFM theory on the atomic scale, the usually assumed qualitative proportionality to the out-of-plane electric field gradient breaks down at small tip–sample separations, and convolution with unknown background force contributions further complicates the analysis. Attempts to address these problems led to the recent development of scanning quantum dot microscopy,^25^ which offers an alternative approach to map the local potential of a surface and adsorbates at high resolution.^26^ While powerful, the technique relies on quantum dot tip functionalization and a dedicated controller, again limiting its broad implementation as yet.

In this work, we were inspired by earlier efforts which use AFM tips functionalized with molecules of different electrostatic character to resolve the electrostatic potential by comparing the characteristic distortions of each tip.^27^ However, the limitations of the approach meant it was unable to provide quantitative accuracy. Here, we offer electrostatic discovery atomic force microscopy (ED-AFM), a machine learning (ML) approach that can predict accurate electrostatic fields directly from a set of standard experimental AFM images. This methodology offers convenient access to the electrostatic potential of molecules adsorbed on surfaces, which will be important in—for example—understanding their catalytic activity, identifying products of on-surface synthesis routes and facilitating chemical identification of functional groups in unknown molecules.

## Results and Discussion

At the heart of the ED-AFM methodology lies a convolutional neural network that is trained to connect input data—a set of constant height AFM images of the frequency shift Δ*f* at different tip–sample distances acquired with two different tip charges—to a map of the electrostatic potential over the molecule (ES map descriptor). The details of the procedure are given in the Methods section.

### Benchmark Systems

**Figure 1:**
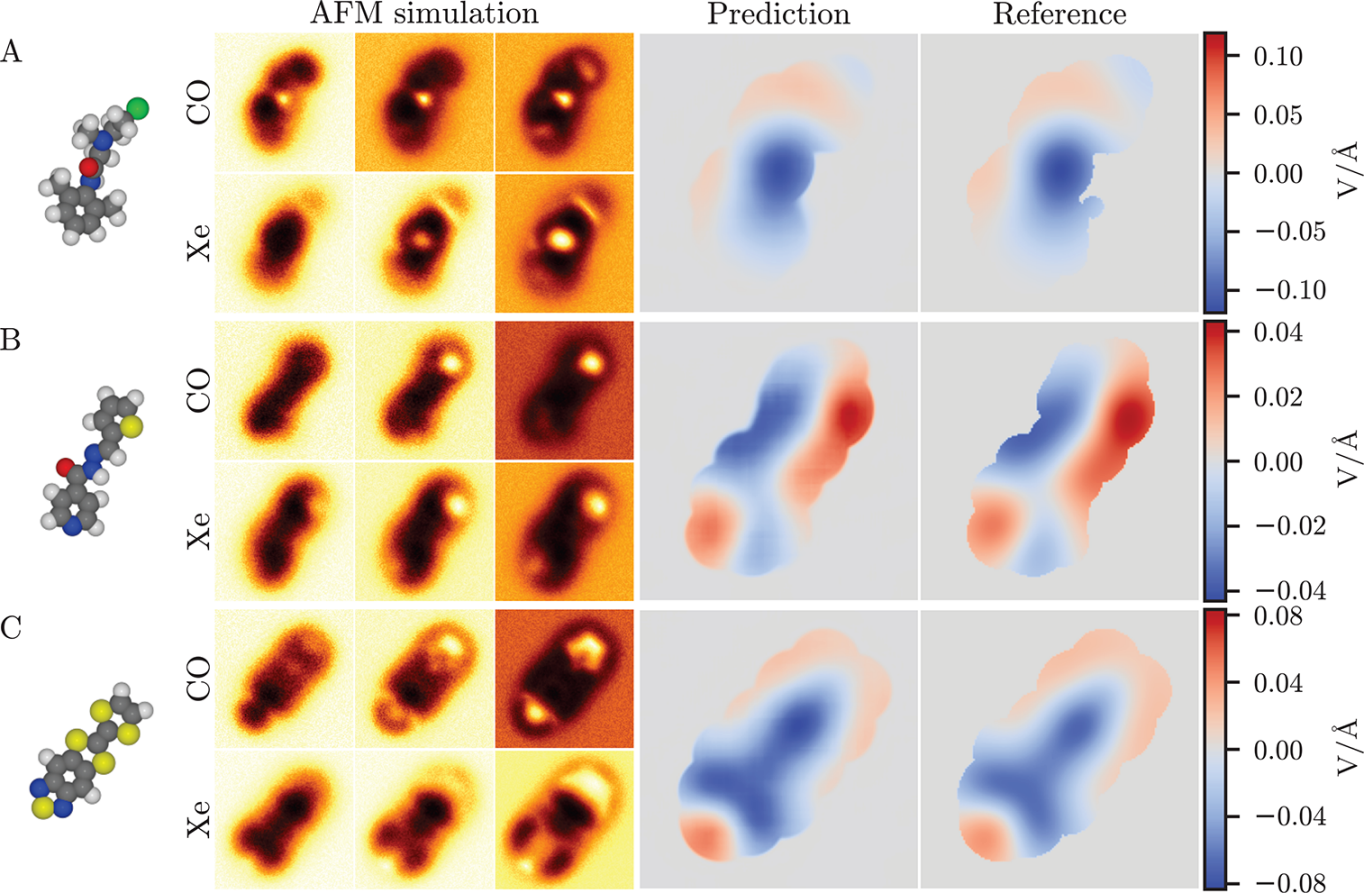
Predictions on simulated AFM images. Predictions are shown for three test systems, (A) N2-(2-chloroethyl)-N-(2,6-dimethylphenyl)-N2-methylglycinamide, (B) 2-[(1E)-2-thienylmethylene]-hydrazide, and (C) tetrathiafulvalene thiadiazole. In each case are shown, from left to right, the 3D structure of the molecule, three out of six input AFM images at different tip–sample distances for both tip functionalizations, and the predicted and reference ES Map descriptors. The color-bar scale for the prediction and the reference is the same on each row.

In order to benchmark the ED-AFM method, we first consider three molecular systems using only simulated data. The first two, “N2-(2-chloroethyl)-N-(2,6-dimethylphenyl)-N2-methyl-glycinamide (NCM)” and “4-pyridinecarboxylic acid, 2-[(1E)-2-thienylmethylene]hydrazide (PTH)” (see Figure 1A,B) were chosen due to the presence of different functional groups and bonds, as well as due to their nonplanar geometry. In the third example, we focus on a charge transfer complex, polar tetrathiafulvalene thiadiazole (TTF-TDZ; see Figure 1C), which was characterized electrostatically in a previous work using KPFM and *ab initio* simulations.^24^ These examples are only considered as free-standing molecules, and therefore the presented orientations and geometries are likely not fully representative of those that the molecules would adopt on a surface.

We consider here the predictions in comparison to the point-charge reference that the model was trained to reproduce. In all cases the match between the predicted and the reference descriptors is generally excellent. The positive and negative regions are predicted in the correct places at a correct magnitude with some small imprecision at the edge regions. For a more quantitative comparison, we consider a relative error metric:
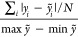
where *y* is the predicted descriptor, *ỹ* is the reference descriptor, and the sum is over the *N* pixels in the descriptor. This is the mean absolute error in the prediction relative to the range of values in the reference. For our three benchmark examples, we find that the relative errors are 1.04% for NCM, 1.79% for PTH, and 2.29% for TTF-TDZ.

### Validation

**Figure 2:**
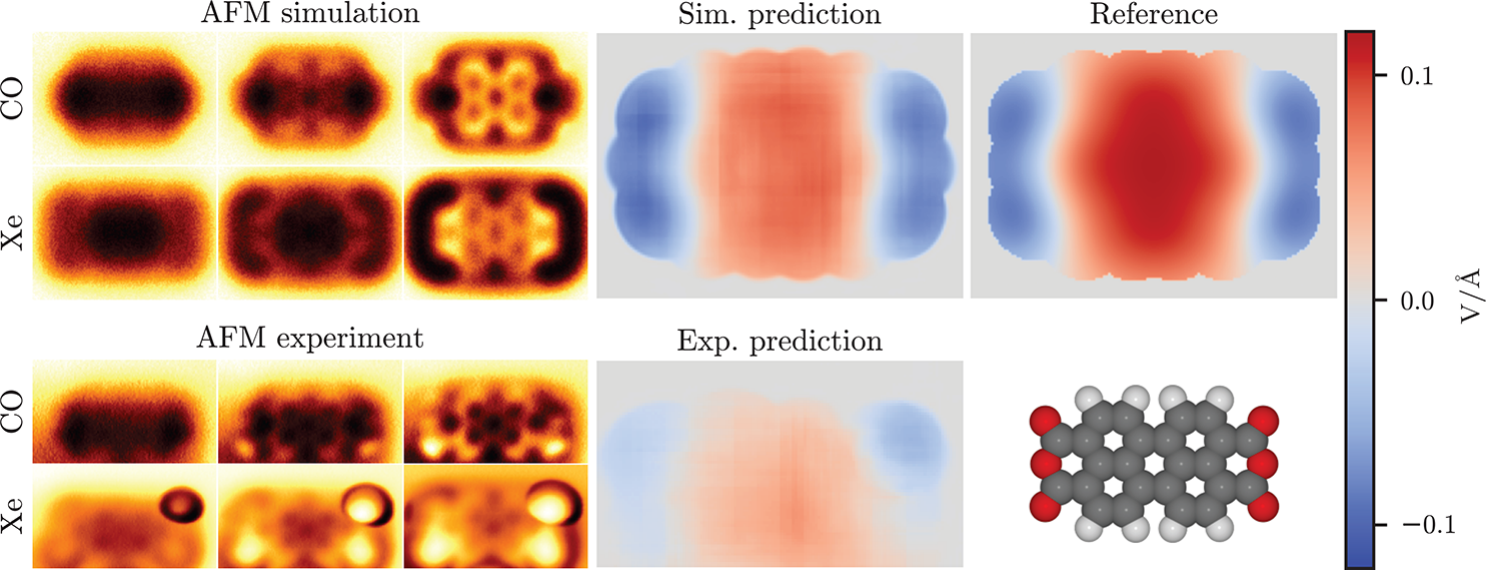
Comparison of simulated and experimental predictions for perylenetetracarboxylic dianhydride. On the left are shown three out of six input AFM images at different tip–sample distances for both tip functionalizations, and on the right are the model predictions for both simulation and experiment and the reference descriptor. Both predictions and the reference are on the same color-bar scale. The molecule geometry used in the simulation is shown on the bottom right.

Clearly, the real test of ED-AFM is with experimental data, and we consider three representative example molecular systems. In our first example, we consider perylenetetracarboxylic dianhydride (PTCDA) on the Cu(111) surface (see Figure 2), a benchmark system in the analysis of molecules on metal surfaces and in characterizing electrostatic interface properties.^26,28,29^ The simulation is done on a free-standing molecule with planar geometry using point charges for electrostatics. In the reference ES Map descriptor, we find that the ends of the molecule with the three oxygens have a negative field, as would be expected by the electronegativity of oxygen, and the field in the middle of the molecule is positive. In line with the previous simulation examples, the prediction based on the simulated AFM images is in good agreement with the reference. The prediction from the experimental AFM images similarly shows a negative field over the ends of the molecule and the positive field in between. This matches well with the reference, except for the somewhat weaker magnitude of the field in the experimental prediction.

We note that in the experimental Xe-AFM images, there appears an abnormally large, bright feature in the upper right corner over one of the oxygens. The origin of this artifact is unclear, but we speculate that it could be due to a difference in charge at that site (see Supporting Information (SI) section “Possible extra electron in PTCDA” for further discussion). Despite this unusual feature in the AFM images, the model prediction does not appear to be greatly disturbed over the corresponding region. Another unusual feature in this set of images is that there is a gradient in the background of the AFM images decreasing from the upper edge toward the lower edge of the images. This is due to the experiment being performed at a slight tilt with respect to the surface. Originally, our model could not handle this feature in the images very well, since such features originating from the surface are normally absent from the simulations that only consider free molecules. After adding artificial gradients in random directions to the simulation images in training, we found that the model began to perform more consistently in the PTCDA experiment. See the SI section “Surface tilt effect on model predictions” for further discussion.

**Figure 3:**
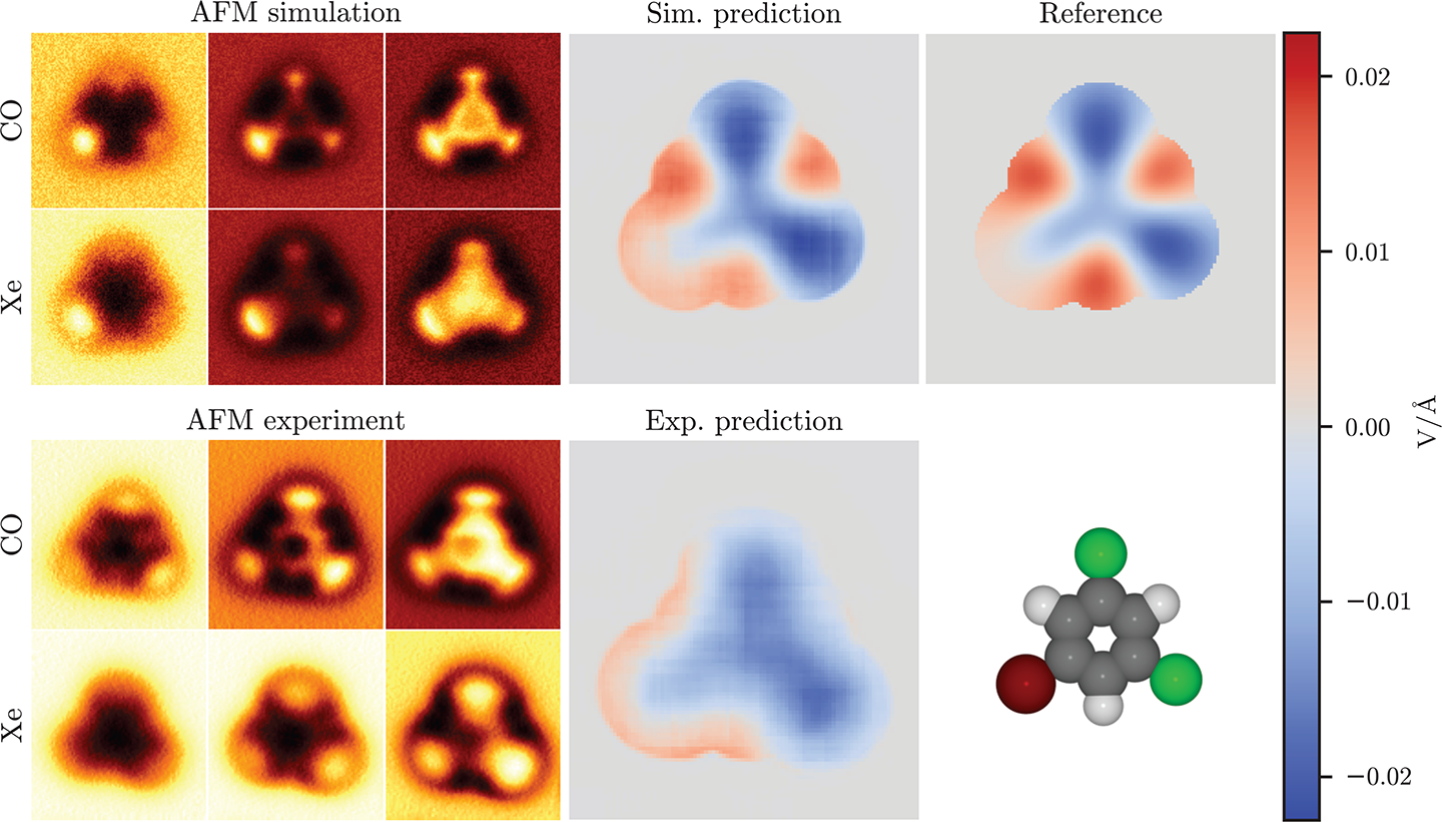
Comparison of simulated and experimental predictions for 1-bromo-3,5-dichlorobenzene. On the left are shown three out of six input AFM images at different tip–sample distances for both tip functionalizations, and on the right are the model predictions for both simulation and experiment and the reference descriptor. Both predictions and the reference are on the same color-bar scale. The molecule geometry used in the simulation is shown on the bottom right.

For our second example, we study 1-bromo-3,5-dichlorobenzene (BCB) on the Cu(111) surface (see Figure 3), a planar molecule with mixed halide functionalization. As with PTCDA, for simulations of this molecule, we consider a completely planar free-standing molecule with point-charge electrostatics. In the reference ES Map descriptor, we find a negative field both over the chlorines and in the middle of the molecule, close to neutral over the bromine, and positive over the hydrogens. Again, the simulation prediction is in good agreement with the reference. In the prediction for the experimental AFM images, we see a region of positive field running along the edge of the lower left part of the molecule, where we suppose the bromine is, and negative field over the other two halides. This matches quite well with the reference, also in the magnitude of the field, except for the missing positive region over the hydrogen opposing the bromine. We note here that the experimental Xe-AFM images have been flipped left-to-right and slightly rotated due to the molecule having rotated between the CO and Xe experiments. The original images can be seen in SI Figure S6.

**Figure 4:**
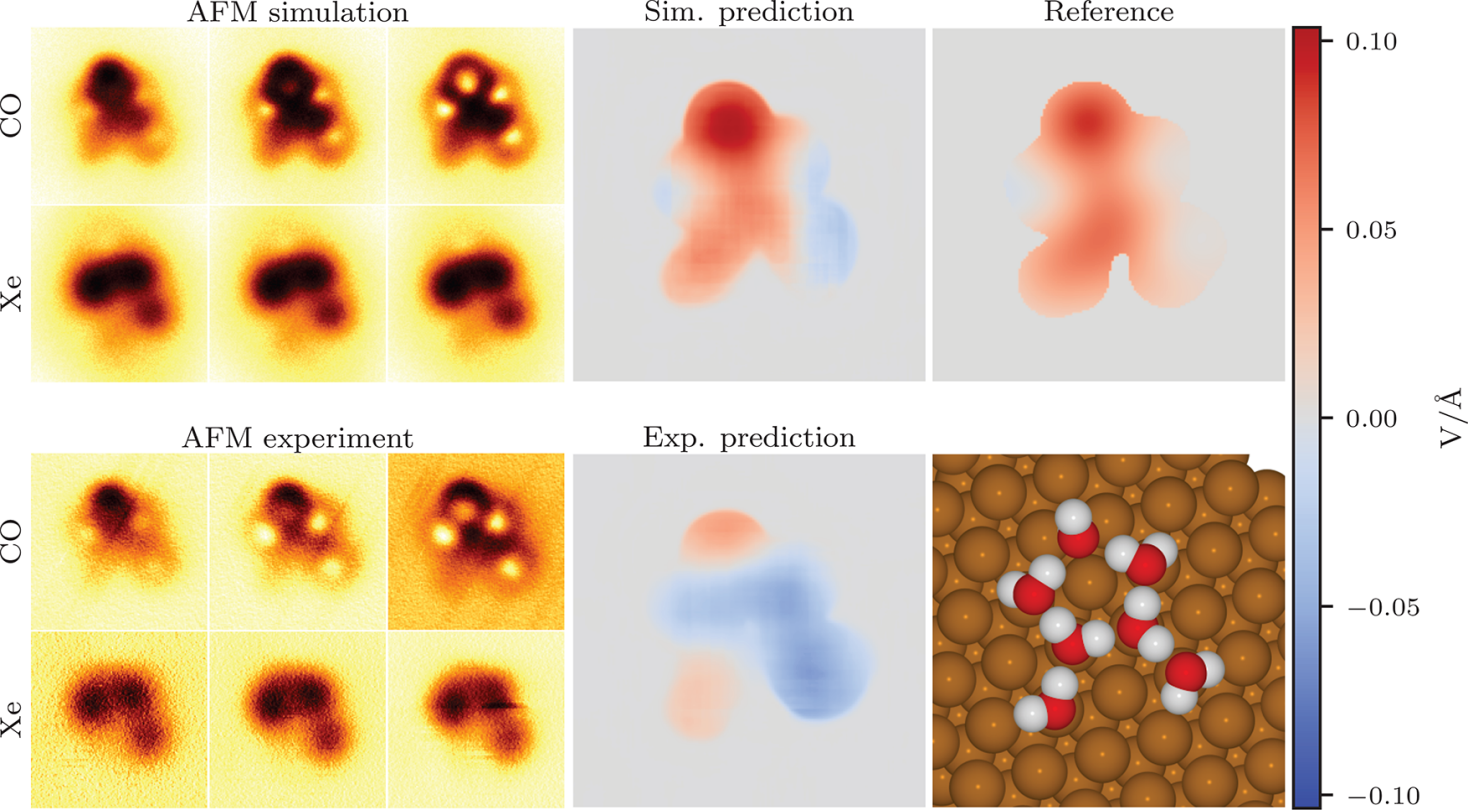
Comparison of simulated and experimental predictions for a water cluster on Cu(111). On the left are shown three out of six input AFM images at different tip–sample distances for both tip functionalizations, and on the right are the model predictions for both simulation and experiment and the reference descriptor. Both predictions and the reference are on the same color-bar scale. The molecule geometry used in the simulation is shown on the bottom right.

Our final example is a cluster of seven water molecules on the Cu(111) surface (see Figure 4). Again, we use point-charge electrostatics, but this time we include the metallic surface, since this configuration of water molecules is only stable on a surface. The seven water molecules form a single five-member ring with two additional molecules forming an incomplete second ring. Similar water clusters with nine and 10 molecules forming two complete rings were previously studied with a combination of DFT calculations and STM experiments.^30^ In our calculations, we find that having the second ring be incomplete results in a better match between the simulated and experimental AFM images.

Here in the reference ES Map descriptor, we find that the field is mostly positive over the whole structure with some neutral regions on the sides. The simulation prediction matches the reference well except for the sides where the prediction is more negative. The match of the experimental prediction with the reference is reasonable, capturing the positive regions at the top part and at the bottom left “leg” of the structure, but it also has a significantly larger negative region in the middle and right side compared to either the simulation prediction or the reference.

Since we have obtained more than the required six height slices for each tip in the experiments, we also consider what happens to the predictions as functions of tip–sample distance for the PTCDA and BCB experiments and find that the predictions stay consistent over small deviations to the distance in either direction (see SI section “Distance dependence”).

### Limitations of the Current Model

Until this point we have been considering only the point-charge electrostatics that the ML model was trained on and found that the model performs well on the simulation examples and the experimental predictions are in fairly good agreement with the ones for simulations. However, the point-charge model of electrostatics has its limitations and in many cases does not perfectly represent the true charge distribution and, by extension, the electric field of the sample. In order to test the validity of the results so far, we performed density functional theory (DFT) calculations using the all-electron density functional theory code FHI-AIMS,^31^ implementing the “tight” basis with the PBE functional^32^ and TS van der Waals^33^ for all the test systems to obtain their Hartree potentials, which can be used for more accurate electrostatics in both the AFM simulations and the reference ES Map descriptors. We first test the Hartree potentials on our three simulation benchmark systems (Figure S7 in SI) and find that the Hartree reference descriptors remain mostly the same as the point-charge ones. The predictions are still qualitatively fairly good and are at least semiquantitative in accuracy. The relative errors are 6.12%, 4.78%, and 7.34% for NCM, PTH, and TTF-TDZ, respectively.

**Figure 5:**
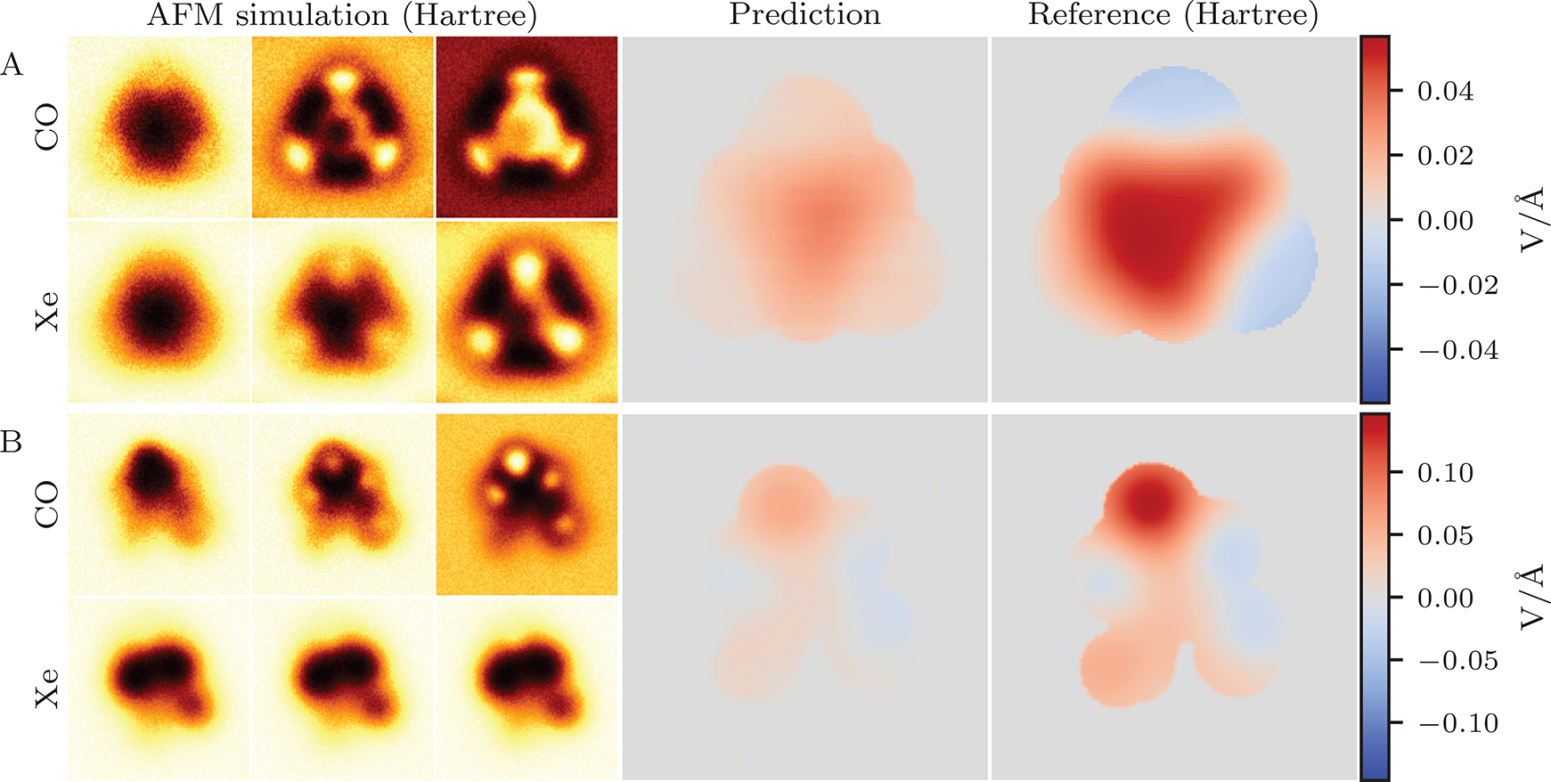
Prediction and reference for on-surface geometry of (A) BCB and (B) the water cluster using the DFT Hartree potential for electrostatics in the AFM simulations and for the reference ES Map descriptor.

Next, we test the Hartree potential on BCB, one of our experimental test systems. For this test, we first relax the geometry on a Cu(111) surface to obtain a more accurate geometry for the molecule. The resulting AFM simulation based on the Hartree potential rather than point charges, the ML prediction, and the reference descriptor are shown in Figure 5A. In the on-surface geometry the bromine is attracted closer to the surface, giving the molecule a slight tilt. This makes the bromine appear less bright in the AFM simulation compared to the planar geometry with point charges, which matches better with the experimental AFM images. The Hartree reference ES Map has similar pattern of field to that of the point-charge reference over the edges of the molecule, but they differ in the middle over the carbon ring where the Hartree reference has a strong positive field instead of a negative field. The prediction on the simulation using the Hartree potential correctly catches this positive field in the middle of the molecule, but it misses the negative field over the chlorines, and the magnitude of the field is overall too small, roughly by a factor of 2. However, the prediction on the experimental AFM images compares more favorably with the ES Map based on point-charge than with DFT/Hartree, even though the Hartree potential from DFT should represent a more accurate description of reality. Note here that it is not *a priori* clear which (if either) ES map the experiment should reproduce: using point charges and the geometry of an isolated molecule is not expected to be the best description of the real BCB molecule on the Cu(111) surface. On the other hand, if we run the AFM simulations based on DFT/Hartree molecular structure and electrostatics, we would not expect the ML to reproduce the ES map reference as the model is trained on point charges. Understanding this in detail requires development of a model trained on out-of-distribution samples using the Hartree potential, a focus of future work.

We also perform the DFT calculation for on-surface PTCDA, and find that in this case the model performs poorly even on the simulated data (Figure S8 in SI). However, establishing accurate geometries for PTCDA on metal surfaces is very challenging,^34^ and indeed, when we perform AFM simulations for our on-surface geometry for PTCDA (SI Figure S8) we find that the simulation shows an asymmetric and much larger contrast between the ends and the middle of the molecule than in the experimental images. This indicates that the real geometry of the molecule is more planar and more symmetric than in our DFT simulation. Therefore, we feel the reference ES Map descriptor for PTCDA obtained from the DFT Hartree potential here does not provide a valid point of comparison with the experimental prediction.

Finally, we test the Hartree potential on the water cluster (Figure 5B) using the same geometry as with the point charges. Compared to BCB, the difference between the point charges and the Hartree potential is less pronounced, both in the simulated AFM images and the reference ES Map. The biggest difference is the appearance of a stronger positive region at the top of the structure and more negative fields on the sides, which takes the reference, at least qualitatively, closer to the ES Map predicted from the experimental AFM images. The prediction on the simulated images using the Hartree potential is qualitatively good but is weaker in magnitude, especially at the positive region at the top. The relatively weaker magnitude of the predictions compared to the reference seems to be the general pattern for all of our cases using the Hartree potential.

## Conclusions

This work demonstrates that ED-AFM offers a method for the rapid prediction of electrostatic properties directly from experimental AFM images. We have shown that these predictions demonstrate quantitative accuracy with minimal computational cost once the machine learning infrastructure is trained. In particular, the comparison between reference electrostatics and ML predictions from simulated AFM data has an error of about 1–2%. However, it is also clear, as for any ML-based approach, that the method cannot predict what it has not learned and it performs poorly in systems where point charge electrostatics are a poor approximations—even for simulated data, the error increases to 5–7%. We will address this in future work: as part of the database generation, we have stored full density matrices with higher-order quantum chemical accuracy,^35,36^ and we are developing methods for their efficient incorporation into the training process. However, we also note that fixed point charges offer decent accuracy in many systems and are routinely used in molecular modeling.^37,38^ The further limitations of ED-AFM lie mainly in the challenges posed for experiments and, in particular, obtaining images of the same system with two different tips. As we have demonstrated, this is feasible on well-defined metal surfaces but can pose problems on less standard samples. This can be at least partially alleviated by developing methods for the autonomous functionalization of the tip in AFM (see, e.g., https://github.com/SINGROUP/Auto-CO-AFM). We note that the two different tips do not need to be CO and Xe, and we have shown, at least for simulated data, that other pairs would work effectively for ED-AFM. Finally, as for any machine learning based approach, the predictions can be confused by unexpected artifacts. We apply a suite of tools to improve robustness (see SI sections “Machine learning” and “AFM Simulations”), and due to the longer range of electrostatic forces, the predictions are less dominated by images at close approach and hence less sensitive to tip-induced relaxations.^39^

Finally, we note that ED-AFM also offers the prospects of application beyond the examples considered here, to any system where electrostatic characterization is of interest. For example, expansion to predictions for assembled layers,^40,41^ defects,^42,43^ and charge dynamics^44^ requires only development of the training data to ensure that it is general enough. The method can also be applied to images of systems where direct simulation is impossible due to size, complexity, or lack of information. Combined with developments in the autonomous functionalization of the tip in SPM,^45^ this promises a future of potential in electrostatics for ED-AFM.

## Methods/Experimental

In general, the adoption of ML methods into materials analysis has seen rapid recent growth,^46−48^ and this has been followed by an equivalent growth in its applications to image analysis in SPM.^45,49−56^ Here, we build upon our ML method for predicting molecular structure from AFM images,^39^ to predict the electrostatic field of the sample molecule.

**Figure 6:**
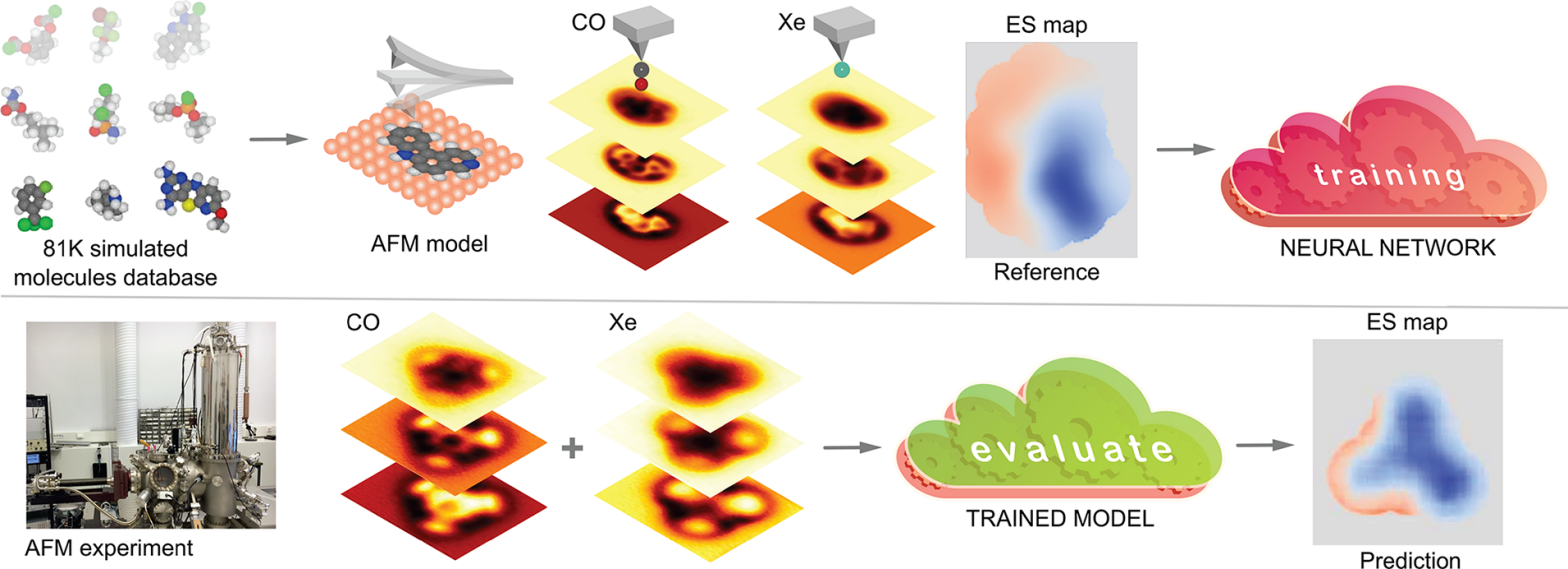
Schematic of the ED-AFM method. We train a neural network that takes two sets of AFM images as input and translates them to the ES Map descriptor, which is the vertical component of the electrostatic field over the sample molecule. The model is trained on simulated sets of input–output pairs calculated from a database of several tens of thousands of molecule geometries. The trained model can then be applied to experimental AFM images to produce a prediction of the sample electric field.

The overall idea of our method is illustrated in Figure 6. We train a deep learning model on a data set of simulated AFM images and reference descriptors based on a large database of molecular geometries, including electrostatics at the level of point charges taken from the associated quantum chemistry calculations.^39^ For more details on how the data set is generated from the geometries, see the section “Data set” in the Supporting Information (SI). The trained ML model can then be used to make predictions with experimental data as input. More specifically, the ML model takes as input two sets of six AFM images of the same sample at different tip–sample distances, imaged with two different functionalizations of the AFM tip. The model, which is an Attention-U-Net-type convolutional neural network^57,58^ (more details in SI section “Machine learning”), translates the AFM images into a descriptor of the imaged sample, which we call the electrostatic (ES) map. The ES Map is defined as the vertical component of the electrostatic field calculated on a constant-height surface 4 Å above the highest atom in the sample. Furthermore, the nonzero region is cut only into the region where the sample molecule of interest is visible in the AFM images, such that the ML model is not asked to predict the field over the background where there are no discernible features present. For a more detailed description of how the ES Map descriptor is generated, see the SI section “ES Map descriptor”.

The use of two sets of AFM images in the input is motivated by the observation that the different distortions in the images obtained with different tip functionalizations are linked to the different electronic charges on the tips.^27^ Thus, given a database of such pairs of images, an ML model should be able to learn what role the electrostatics play in the formation of the images and separate the electrostatic contribution from other forces that contribute to the images. Here for the tip functionalizations, we use CO, which has a slightly negative charge, and Xe, which has a somewhat positive charge. Other choices for functionalization are possible, and we investigate the alternative tip combinations of Cl–CO and Cl–Xe on simulated data in the SI section “Other tip combinations”. We also tried training a model using images of only a single tip functionalization with CO but found the results to be less robust (see SI section “Single-channel measurements”). As in our previous work,^39^ we train the model using simulated AFM images and validate the model using both simulated and experimental AFM images. Our implementation of the model in Pytorch^59^ with pretrained weights can be found at https://github.com/SINGROUP/ED-AFM.

The manuscript was previously submitted to a preprint server.^60^

## Supporting Information Available

Details of the machine learning models and training data, including investigation of the sensitivity of the predictions to tip–surface distance, noise, various simulation parameters, and the number of channels used in experiments, and also expanded discussion of the AFM simulations and experiments themselves (PDF)
