# Correction to Electrostatic Discovery Atomic Force Microscopy

**DOI:** 10.1021/acsnano.2c08130

**Published:** 2022-09-14

**Authors:** Niko Oinonen, Chen Xu, Benjamin Alldritt, Prokop Hapala, Filippo Federici Canova, Fedor Urtev, Shuning Cai, Ondřej Krejčí, Juho Kannala, Peter Liljeroth, Adam S. Foster

**Affiliations:** † Department of Applied Physics, Aalto University, 00076 Aalto, Espoo, Finland; ‡ FZU - Institute of Physics of the Czech Academy of Sciences, 182 21 Prague 8, Czechia; § Nanolayers Research Computing Ltd., London N12 OHL, United Kingdom; ∥ Department of Computer Science, Aalto University, 00076 Aalto, Espoo, Finland; ⊥ WPI Nano Life Science Institute (WPI-NanoLSI), Kanazawa University, Kakuma-machi, Kanazawa 920-1192, Japan

Recognizing his contribution to the early code development, we are amending the author list to include our collaborator Prokop Hapala as a co-author, who could not be reached at the time of the original article submission.

