# Supplementary material for: Electrostatic Discovery Atomic Force Microscopy
Source: ACS Nano. 2021 Nov 22;16(1):89–97. doi: 10.1021/acsnano.1c06840 (PMC8793147; doi:10.1021/acsnano.1c06840)
Supplement: Supplementary file 1 — nn1c06840_si_001.pdf [file nn1c06840_si_001.pdf]

Supplementary Information: Electrostatic Discovery Atomic Force Microscopy

Niko Oinonen,[1*], Chen Xu,[1*], Benjamin Alldritt,[1*]
Filippo Federici Canova,[1,2] Fedor Urtev,[1,3] Shuning Cai,[1] Ondřej Krejčí,[1]
Juho Kannala,[3] Peter Liljeroth[1†] and Adam S. Foster[1,4†]

[1]Department of Applied Physics, Aalto University, 00076 Aalto, Helsinki, Finland
[2]Nanolayers Research Computing Ltd, London N12 0HL, United Kingdom
[3]Department of Computer Science, Aalto University, 00076 Aalto, Helsinki, Finland
[4]WPI Nano Life Science Institute (WPI-NanoLSI), Kanazawa University, Kakuma-machi, Kanazawa 920-1192, Japan

[*]These authors contributed equally.
[†]To whom correspondence should be addressed;
E-mail: peter.liljeroth@aalto.fi; adam.foster@aalto.fi.

Methods

Machine learning

The core of our model has the structure of the U-Net [1], where the feature maps first enter an encoder which down-samples them with pooling layers and then enter a decoder which up-samples them back to the original size, with skip connections between the layers of matching size in the encoder and decoder. In addition to the different number of channels and layers, the main difference to the original U-Net is that we start the network with 3D convolutions and then change to 2D convolutions in the middle, and we use Attention-Gate (AG) [2] layers in the skip connections. Around the core, we have the input stage which merges the two input sets of AFM images, and the output stage which outputs the ES Map descriptor. All of the convolutional layers use replicate padding, and, except for the output layer, all convolutional layers are followed by LeakyReLU activations [3] with negative slope of 0.1.

The model architecture, along with the shapes of the layers assuming 128×128 lateral input size, are illustrated in Fig. S1. At the start, the two inputs are fed into their own blocks of two 3D convolutions with 32 channels. The outputs from these are concatenated together in the channel dimension and then fed into the encoder. The encoder consists of three blocks of 3D convolutions and poolings. In each block, there are three 3D convolutions, with 48, 96, and 192 channels in each block, respectively, and the poolings are AvgPool layers. The poolings all have pool regions of $2 \times 2 \times 2$, but the middle pooling has a stride of $2 \times 2 \times 1$, so that the size of the feature map in z-direction is only reduced by 1. After the last pooling layer, the 3D feature maps are transformed into 2D feature maps by concatenating the remaining z-layers of the 3D feature maps into channels of the resulting 2D feature maps. The middle section between the encoder and the decoder has a block of three 2D convolutions with 512 channels. The decoder has three up-sampling stages corresponding to the three down-sampling stages of

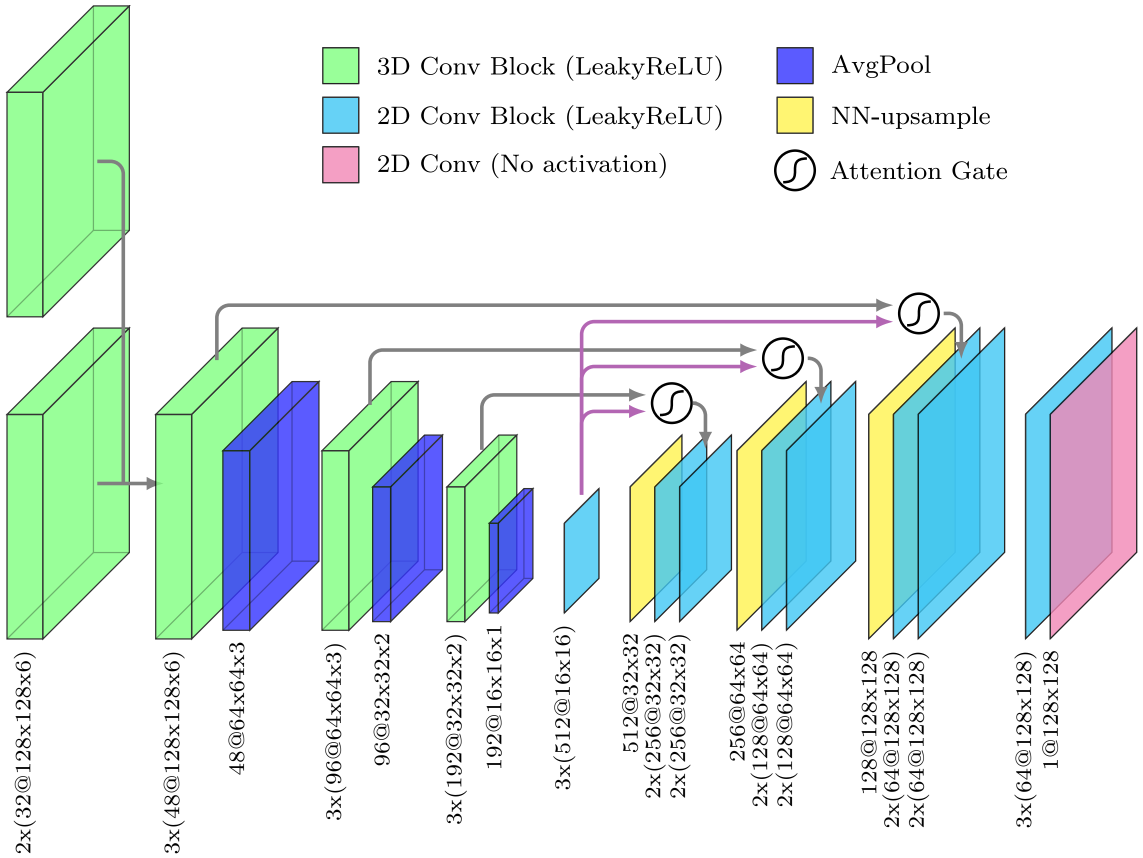

Figure S1: Schematic illustration of the model architecture. Below each layer or block of layers, the output shape of the layer is reported in the format (number of channels)@(feature map size) assuming a 128×128 lateral input size, and in the blocks the first number indicates the number of layers of that shape in the block. Here, NN = nearest neighbour.

the encoder. At each stage there is first a nearest-neighbour up-sampling followed by a block of two 2D convolutions. Then the skip connection from the corresponding stage of the encoder is passed through the AG and is concatenated as additional channels to the input of a second block of 2D convolutions. The convolution blocks in the decoder stages have 256, 128, and 64 channels. After the decoder, the model has one more 2D convolution block with three 2D convolutions and 64 channels, and one more 2D convolution with a single channel to output the ES Map descriptor. The total number of parameters in the model is 15,604,900.

In the proposed architecture we implemented AGs [2] on the skip connections from the

encoder to the decoder. Based on a specific task, AGs can suppress irrelevant and highlight useful parts in inputs. An AG architecture is illustrated in Fig. S2. It has two inputs: a set of feature maps from 3D convolution blocks in encoder flattened into 2D feature maps (x) and a query (q) – feature maps from the last 2D convolution layer in the middle part of the model. For a 128×128 lateral input size, the skip connections have the following shapes: 288@128x128, 288@64x64, 384@32x32 and the query shape is 512@16x16. After an interpolation of q to match the shape of x, both x and q are passed through independent 2D convolution layers with ReLU activations and then combined together by channel-wise summation. The feature maps are then passed through a 2D convolution with a Softmax activation to create a single-channel map of attention coefficients – the attention map. Finally, the attention map is mixed with the skip connection by element-wise multiplication in each channel. Due to the construction with a Softmax activation, the AG learns to highlight the most relevant regions in the input without explicitly being trained to do so.

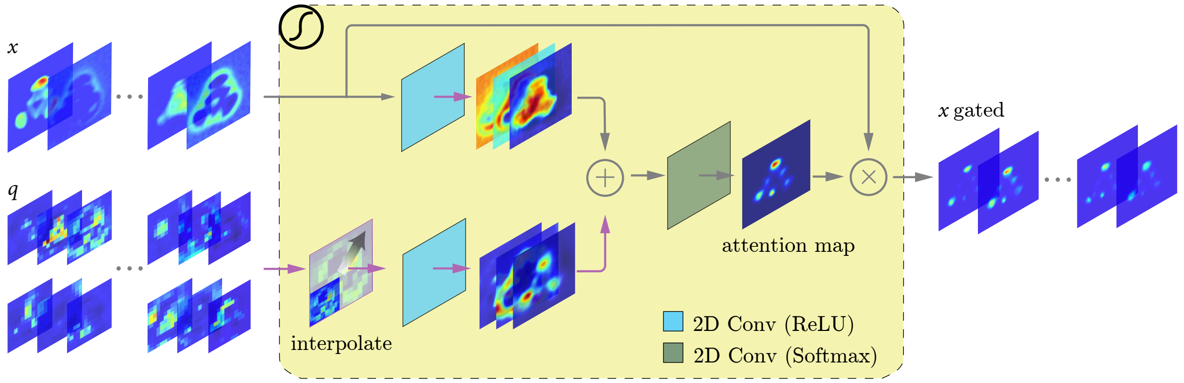

Figure S2: Schematic illustration of the Attention Gate (AG) using the BCB molecule as an example. Randomly picked features maps in inputs and outputs are presented. AG operates with 2 inputs: skip connection feature maps (input x) together with compressed representation at the end of the encoder (query q). Since all three skip connections have different sizes than the query, q is interpolated to match the size of x. Both x and q are passed through 2D convolutions with ReLU activation, and then they are summed together and the result is passed through a 2D convolution layer with Softmax activation to produce the attention map. The attention map is finally multiplied pixel-wise with the skip connection features maps to produce the gated output of the AG layer.

Our objective function is the mean squared error

$$\mathrm{MSE}(y, \tilde{y}) = \frac{1}{N}\sum_{i=0}^{N-1}(y_i - \tilde{y}_i)^2, \tag{1}$$

where y is the predicted ES Map, $\tilde{y}$ is the reference ES Map, and the sum is over the N pixels. For reference, the losses on the final trained model are 2.17×10^{-5} on the training set, 2.49×10^{-5} on the validation set, and 2.47×10^{-5} on the test set. The parameters are optimized using the Adaptive Moment Estimation (Adam) optimizer [4], with learning rate 10^{-4} and the default values of $\beta_1 = 0.9$ and $\beta_2 = 0.999$ for the moment decay parameters. Additionally, we use a learning rate decay, where on each iteration i, the initial learning rate is multiplied by a factor

$$\frac{1}{1 + 10^{-5} \cdot i}. \tag{2}$$

The training set has a total of 6000 batches with 30 samples each, and the model is trained for a total of 50 epochs. The dataset is described in more detail below in Sec. ”Dataset”.

During training, we preprocess the samples in several ways before they enter the model. The samples are normalized by subtracting the mean and dividing by the standard deviation per each height-layer in the AFM image stack. For regularization, we randomly add to each sample noise, pixel shifts, cutouts, and additive background gradient planes, and the samples are randomly rotated, flipped, and cropped. The noise augmentation is discussed below in Sec. ”Noise amplitude distributions”. The pixel shifts are applied independently to each layer in the AFM image stack, such that the pixel values roll over the borders. The maximum shift between adjacent slices in the AFM image stack is 2% of the image size and maximum total shift is 4% of the image size. The cutouts randomly erase an area of the input image. The erased area for each cutout is at most 1% of the total area of the image and has a maximum aspect ratio of 1 to 10. A maximum of five cutouts are added to each image with 20% probability for each one. For details of the background gradient augmentation, see below Sec. ”Surface tilt effect on model predictions”. The original samples generated in a size of 192×192 are rotated to a random

angle by bicubic interpolation, flipped up-down with 50% probability, and then cropped to size 128×128 to get rid of any empty pixels in the corners. The images are then further cropped to a random position at random size of at minimum 75% of the original size and a random aspect ratio of at most 1.25 in either direction.

The training samples are all generated on a $24 \times 24\ \mathring{A}^2$ frame discretized on a 192×192 grid, and the molecule is always in the center of the frame. Since the model is trained on this specific pixel density of $24\,\mathring{A}/192\,\mathrm{pixels} = 0.125\,\mathring{A}/\mathrm{pixel}$, the experimental images are resized to match this resolution before entering the model. Additionally, we always crop the images into multiples of 8 pixels in each dimension in order to keep the dimensions consistent over the pooling and upsampling layers in the model (three halvings = 1/8 image size). The experimental images are also normalized in the same way as the simulated training samples.

Distance randomization

In AFM experiments, it is often difficult to know the exact distance between the tip and the sample, and the distance range where the tip-sample interaction is stable differs between samples. These facts mean that the range of distances available in AFM images is variable. In order to be robust against varying tip-sample distance, we randomize it during the generation of the training simulation samples within a $0.5\,\mathring{A}$ window. Here we have to take into account the additional factor of the second tip. It is unclear whether the ML model would benefit from having the two tips at the same distance from the sample or if it would also work if the tips are not aligned.

In order to test this, we train the ML model with two differently generated datasets, one with matched tip distance where the tip-sample distance is the same for the two tips for the same training sample, and one with independently randomized tips. We then test how the MSE loss behaves for the two differently trained models as a function of the tip-sample distance on

a subset of 3000 samples from the test set (Fig. S3A,B). For matched tip distances on the test samples (Fig. S3A), we find that both models have almost a flat loss curve within the window of distances used in the training, but outside of that window the loss starts to increase. The increase in loss is especially sharp on the side of smaller distances. We discuss why too close distances are undesirable at more length in the context of the experimental predictions below in Sec. ”Distance dependence”. In these results the difference between the matched and independent tips is small, with possibly a small advantage in favor of the matched tips. However, when we do the test such that the CO-tip is held at constant height and the Xe tip is shifted, the difference in performance becomes very apparent. The model trained on matched tips does well for the zero-shift where the tip distances are matched, but when the Xe distance is varied the loss becomes significantly bigger, by more than a order magnitude even within the training window of distances. This is in contrast with the model trained with independently randomized tips, which has similarly flat loss curve as in the first test. Clearly, any small disadvantage for the independent tip randomization in the first test is worth the trade-off for significantly improved stability when the tip distances are not exactly matched.

Noise amplitude distributions

Experimental AFM images always have some level of noise present in the values of the pixels. In order to be robust against noise in the input images, we add noise with random uniform distribution to the simulated images during training. Since the noise is independent between training epochs, this also serves as a type of regularizing augmentation that reduces overfitting of the model. The generated noise is multiplied by the range of the values ($\max - \min$) in the sample before the addition operation to keep the level of the noise consistent between the samples. We test here three different ways of choosing the amplitude of the noise: constant amplitude, uniform random amplitude, and normally distributed amplitude. For the normal

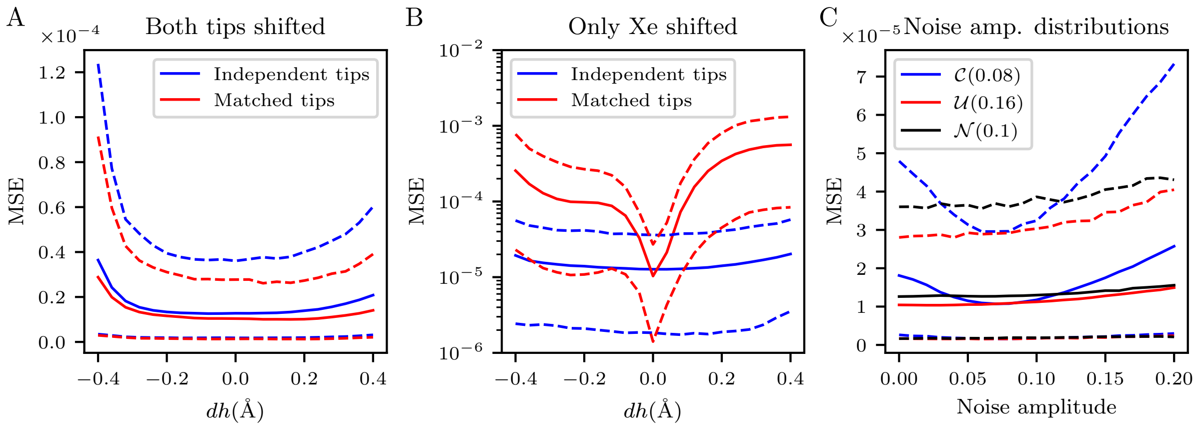

Figure S3: Loss statistics with different randomizations of the tip-sample distance and the noise amplitude on a subset of the test set. (A, B) The MSE loss as a function of tip-sample distance offset dh with (A) both tips offset and (B) only Xe offset for two models trained with independently randomized and jointly randomized distance for the two tips. Here $dh = 0\,\text{Å}$ represents the average distance used in the training. (C) The MSE loss as a function of noise amplitude for three different models trained with constant ($\mathcal{C}$), uniform random ($\mathcal{U}$), and normally distributed ($\mathcal{N}$) noise amplitudes. In all plots, the solid lines represent the mean loss, and the dashed lines represent the 5th and 95th percentiles, so that 90% of the losses are contained within the region enclosed by the dashed lines of the same color.

distribution, we use the absolute value of the generated value as the amplitude, and we choose the standard deviation of the normal distribution to be 0.1. This gives the noise amplitude an expected value of $\sim$$0.08$. To keep the average level of the noise consistent between the tests, for the constant amplitude we choose the value 0.08, and for the uniform random amplitude we choose the range $[0, 0.16]$.

We test these three differently trained models on a subset of 3000 samples from the test set. The average MSE loss on these test samples as a function of the noise amplitude is presented in Fig. S3C. The most striking feature here is the difference between the constant amplitude model and the random amplitude models. The model trained with the constant amplitude does the best on the amplitude of the noise that it was trained on and has worse loss for all other amplitudes, including the zero-amplitude without any noise. This is saying that for this model clean images are harder to interpret than noisy ones, clearly an undesired behaviour. In contrast, the models

trained with random noise amplitude have much flatter loss curves, with the uniform random amplitude having a small advantage over the normally distributed one. Further tests would be needed to determine what is the optimal distribution for the random noise amplitude, but it is clear that random amplitude for the noise is better than constant amplitude. For the training of the model used for the predictions in the main article, we used the normally distributed amplitude.

Dataset

Our model training is based on a database of 81086 molecular geometries containing the elements H, C, N, O, F, Si, P, S, Cl, and Br. The distribution of the elements in the molecules is shown in Table T1. Here we can see that the distribution is not even: H and C are contained in almost every molecule with N and O being very common as well, but the rest of the elements are significantly less common. In our previous work on molecule structural prediction from AFM images we used a simple criterion with a fixed number of rotations for each molecule to choose the molecule orientations for the samples [5]. This lead to an overemphasis on the more common elements, especially H, in the dataset. Here we have chosen the rotations for the molecules more carefully in order to make the element distribution more even in the dataset.

For choosing the rotations, we want to consider what elements are close to the surface of the molecule, so that those atoms could possibly be seen in the AFM images. To this end, we compute the convex hull of the molecule, yielding us sets of three points that define planes on the surface of the molecule. We consider each one of these planes in turn and include the rotation corresponding to the plane probabilistically based on the elements close to the plane, choosing the probabilities such that the rarer elements are emphasized. An element is considered to be close to the plane if an atom with that element is within 0.7 Å of the plane. To counter any bias that using the convex hull planes may incur, we also choose completely random rotations of the

molecules, which are again included probabilistically emphasizing the rarer elements. Finally, we noted that the database does not contain many completely flat geometries, so we include any rotations of the molecules that contain a planar segment, which we define as a plane on the surface of the molecule which contains at least 10 atoms within 0.1 Å of the plane. In order not to have overlap between the rotations, no rotations within $5°$ of each other for the same molecule are included.

Using this procedure, we generate a total of 235554 different orientations of the molecules, which we divide into training, validation, and test sets as 180000, 20000, and 35554 samples, respectively. We take care not to include any of our test molecules in the training or validation sets. The distribution of the elements contained in the final chosen rotations based on the 0.7 Å criterion is shown in Table T1. H and C are still the most common elements, and this is natural, since any orientation where one of other elements is seen, likely H and/or C is also there. The occurrence of the rest of the elements is now more even, except for Si and P, which are mostly only contained inside the molecules and therefore are not often seen close to the surface.

Element	% of molecules in database	% of chosen rotations
H	99.3	87.3
C	99.6	49.8
N	60.8	24.8
O	76.5	29.5
F	5.4	16.5
Si	1.5	0.2
P	3.1	1.2
S	15.0	23.4
Cl	13.7	28.6
Br	3.1	16.4

Table T1: Distribution of different elements contained in the molecules in our database and in the rotations of the molecules that we chose. For the chosen rotations an element is included in the count if it is contained in the region up to 0.7 Å below the top-most atom in the molecule.

ES Map descriptor

The ES Map descriptor is the z-component of the ES field originating from the charges in the sample molecule, calculated at a constant-height surface $4\,\text{Å}$ above the top-most atom in the molecule, and then cut to be non-zero only in the region occupied by the molecule. This process is illustrated in Fig. S4. We define the z-direction to be parallel to the oscillation direction of the AFM probe, which is perpendicular to the hypothetical surface which the molecule would be sitting on, and the positive z-direction is pointing away from the surface (out of the page in the figures here). We find the highest z-coordinate of a center of an atom in the molecule and then add $4\,\text{Å}$ to that value to define the z-coordinate of the constant-height surface of the ES Map. The xy-coordinates of the pixels form a grid corresponding to the matching pixel coordinates in the AFM images. Then for a given pixel $\mathbf{R}_{ij} = (x_i, y_j, z)$ the value of the pixel is

$$E_\mathrm{z}(\mathbf{R}_{ij}) = k_\mathrm{e} \sum_{k=1}^{n} \frac{q_k(\mathbf{R}_{ij} - \mathbf{r}_k) \cdot \hat{\mathbf{z}}}{|\mathbf{R}_{ij} - \mathbf{r}_k|^3}, \tag{3}$$

where k_e is the Coulomb constant, q_k is the charge and $\mathbf{r}_k$ is the coordinate of the kth atom in the molecule, n is the number of atoms in the molecule, and $\hat{\mathbf{z}}$ is a unit vector in the z-direction. For restricting the non-zero area, we use the vdW-Spheres descriptor, which we introduced in our previous work [5]. We modify the descriptor here by adding a constant $1\,\text{Å}$ to the vdW radii of the atoms and restrict the deepest coordinate to be $2\,\text{Å}$ below the top coordinate. We then turn the vdW-Spheres descriptor into a binary mask by setting the background values to 0 and all other values to 1. This mask is then multiplied pixel-wise with the E_z-values computed earlier to produce the final pixel values of the ES Map descriptor.

AFM Simulations

We simulate AFM images using the probe particle model [6]. The procedure for generating the training samples is explained in our previous work [5]. Here we additionally do simulations

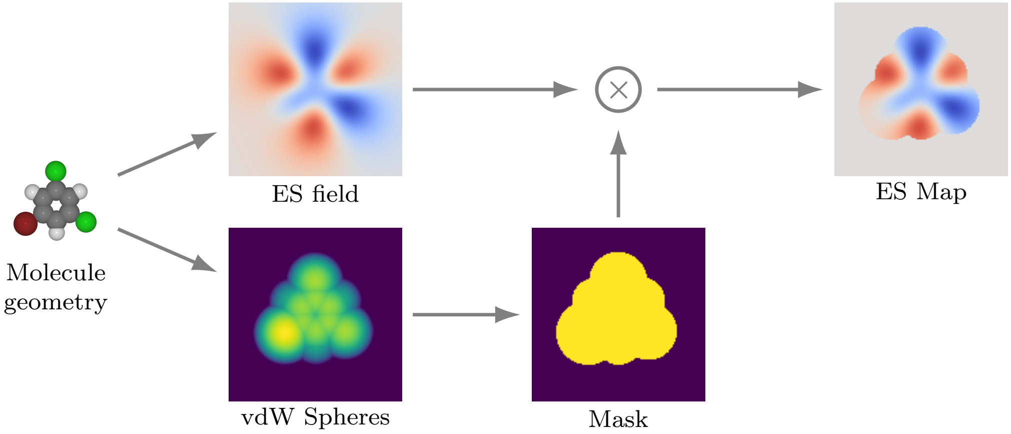

Figure S4: Schematic illustration of the process for generating the ES Map descriptor using the BCB molecule as an example. The molecule geometry is used to compute both the ES field and the vdW-spheres descriptor. The vdW-Spheres descriptor gets turned into a binary mask which is then multiplied pixel-wise with the ES field to produce the ES Map descriptor.

with the Xe and Cl probe-particle tips. The lateral spring constants we use for the probe particles are $0.25\,\mathrm{N/m}$ for CO and Xe, and $0.5\,\mathrm{N/m}$ for Cl. The radial spring constant is $30\,\mathrm{N/m}$ in all cases. The CO and Xe tip charges are modelled as quadrupoles with quadrupole moments of $-0.1\,\mathrm{e}\times\mathrm{Å}^2$ and $0.3\,\mathrm{e}\times\mathrm{Å}^2$, respectively, and the Cl tip charge is modelled as a monopole with a charge of $-0.3\,\mathrm{e}$, where e is the elementary charge. The Lennard-Jones parameters for each atom type contained in our database of molecules are listed in Table T2. To regularize the model and make it more robust, we randomize the tip-sample distance in the simulations within a $0.5\,\mathrm{Å}$ window (see Sec. "Distance randomization" above for more details). Additionally, the lateral equilibrium position of the probe particle is randomized within a disk of radius $0.5\,\mathrm{Å}$.

Sensitivity of predictions to spring constant values

We use fixed values for the lateral and radial spring constants in the AFM simulations in the training set. Since the tip condition can vary between AFM experiments, it is worth considering how sensitive the simulation and the predictions are to the chosen spring constant values. To this end, we run simulations on a subset of the test set varying the spring constant values in

a range of $0.20 \ldots 0.30$ N/m for the lateral spring constant k_{lat}, and $20 \ldots 40$ N/m for the radial spring constant k_{rad}. On visual inspection of the simulated AFM images, for the radial spring constant there is no discernible difference between the different values in the chosen range. For the lateral spring constant, the differences are small, but can still be observed as a gradual change to a slightly sharper contrast in the close range with higher values of the spring constant. To quantify the sensitivity of the model predictions, we run the predictions for the simulated images and record the MSE loss as a function of the spring constant values (Fig. S5). The result is in line with the visual inspection of the AFM images: for the radial spring constant there is no significant difference in the loss values with different k_{rad} values, and for the lateral spring constant the loss increases smoothly when deviating from k_{lat} value used in the training. The loss increases more with decreasing k_{lat}, reaching a value roughly 3 times the minimum loss at $k_{\text{lat}} = 0.25\,\text{N/m}$. Keeping in mind that the MSE loss emphasizes outliers, this does still not correspond to a very large decrease in average performance. However, the result does show that the lateral spring constant is a parameter that could be worth randomizing during the training in the future to be more robust against changes in the tip condition.

Element	R_{ii}[Å]	E_{ii}[eV]
H	1.4870	0.000681
C	1.9080	0.003729
N	1.7800	0.007372
O	1.6612	0.009106
F	1.7500	0.002645
Si	1.9000	0.025490
P	2.1000	0.008673
S	2.0000	0.010841
Cl	1.9480	0.011491
Br	2.2200	0.013876
Xe	2.1815	0.024344

Table T2: Lennard-Jones parameters used in the probe particle simulations.

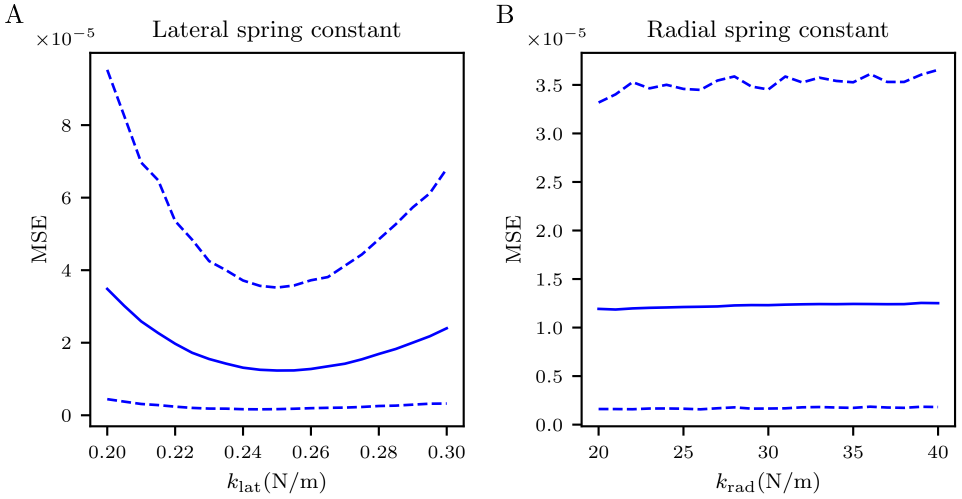

Figure S5: Loss statistics for different values of the spring constants used in the AFM simulation for a subset of the test set. The plots show the MSE loss as a function of the value of (A) the lateral spring constant $k_{\rm lat}$ and (B) the radial spring constant $k_{\rm rad}$. The spring constants are altered for both the CO and the Xe tips. In both plots, the solid lines represent the mean loss, and the dashed lines represent the 5th and 95th percentiles, so that 90% of the losses are contained within the region enclosed by the dashed lines.

Experimental

The AFM images were taken on a combined non-contact AFM/STM system (CreaTec) with a commercial qPlus sensor with a Pt/Ir tip, operating at T $\approx$ 5K in ultrahigh vacuum at a pressure of ~1 × 10^{-10} mbar. The qPlus sensor had a resonance frequency of $f_0 \approx$ 30046 Hz, a quality factor Q = 67714, and was always operating with an oscillation amplitude of A = 50 pm.

The Cu(111) substrate (MaTeck) was prepared by repeated Ne^+ sputtering with a beam energy of 750 eV and ion current of 20 µA for 15 min followed by annealing at 520~550°C for 5 min. A flat Cu (111) surface with large terrace and minimum amount of impurities was often obtained within 3 cycles. The 1-Bromo-3,5-dichlorobenzene molecules (Sigma-Aldrich; purity 98%) were deposited onto the substrate at ~5 K through a variable leak valve **1** at a chamber pressure of 1 × 10^{-6} mbar for 30 seconds. Then the CO molecules (Praxair; purity 99.997%)

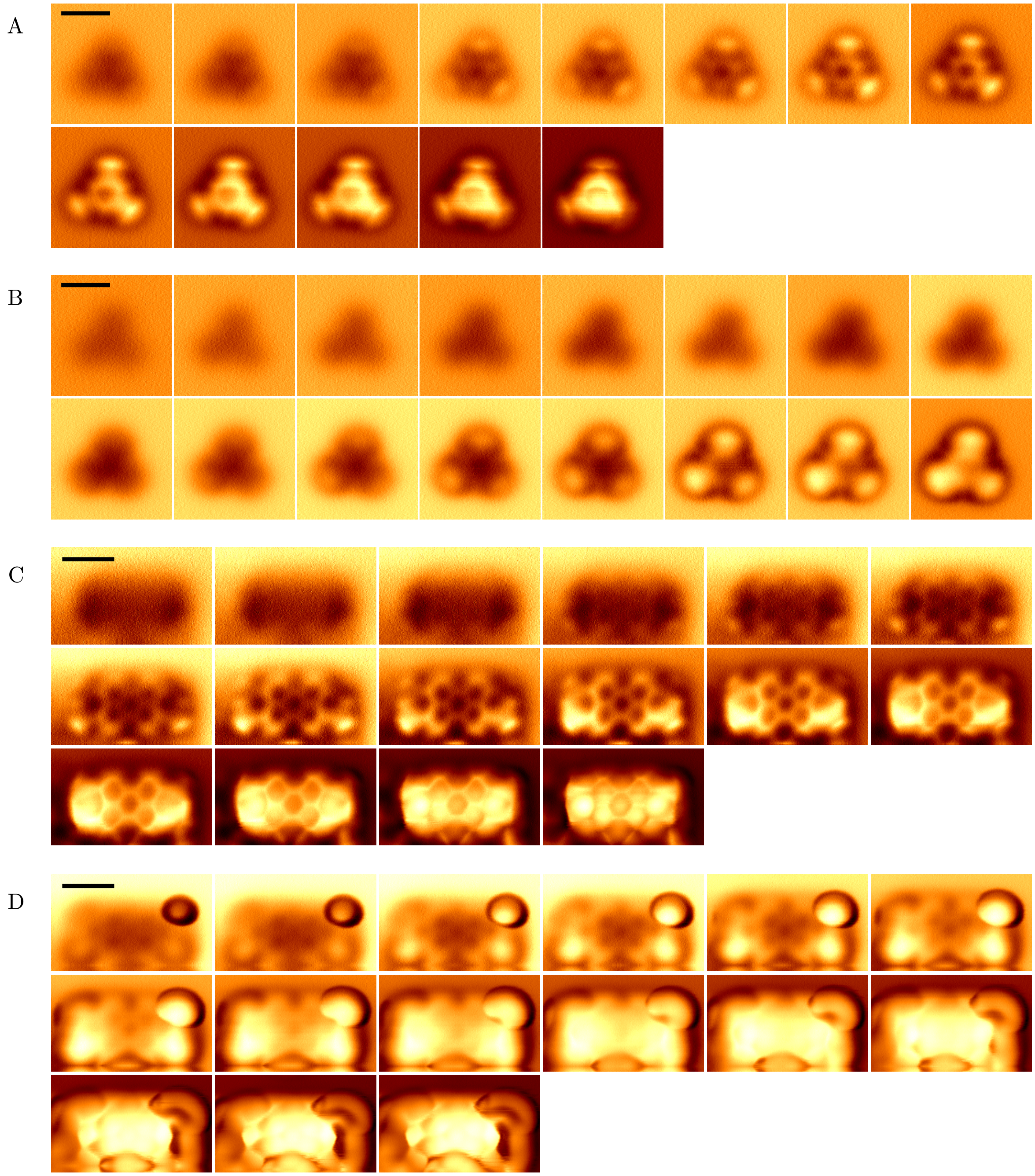

Figure S6: Full sets of experimental AFM images for (A) BCB (CO), (B) BCB (Xe), (C) PTCDA (CO), and (D) PTCDA (Xe). The scale bars are 5 Å long.

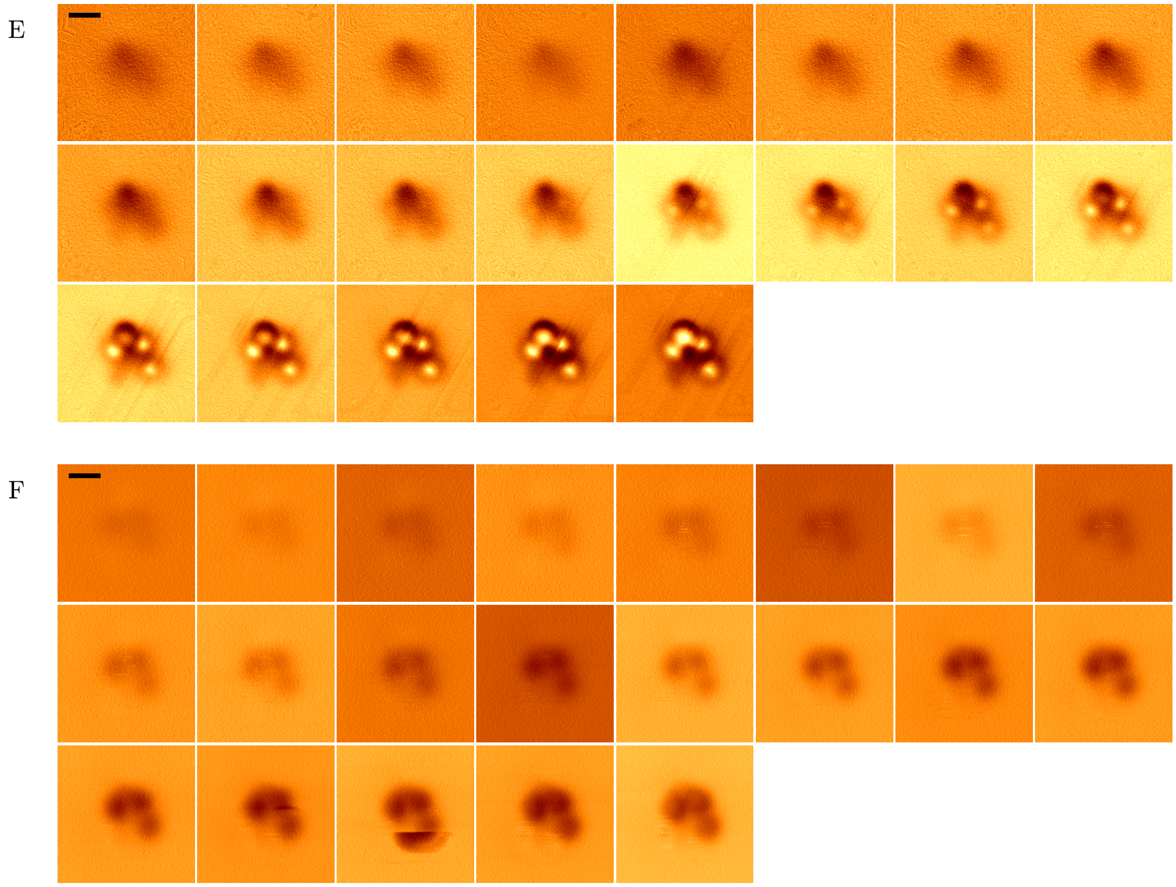

Figure S6: (Continued) Full sets of experimental AFM images for (E) Water (CO), (F) Water (Xe). The scale bars are 5 Å long.

were deposited onto the substrate at 5 K through a variable leak valve **2** at a chamber pressure of 1 × 10^{-6} mbar for less than 5 seconds. Finally, the Xe atoms (Fluka; purity 99.995%) were deposited onto the substrate at 5 K through the variable leak valve **1** at a chamber pressure of 1 × 10^{-6} mbar for 30 seconds.

Tip conditioning was usually performed by controlled contact with the Cu substrate and/or by applying a 1 second voltage pulse of 3~10 V, both with feedback turned off. The tip was deemed as good when a symmetric contact mark was observed as well as a reasonably resolution of the organic molecules was achieved. The tip apex was believed to be covered with Cu atoms

after these operations.

The constant height AFM images were taken with metal tips functionalized with a single CO molecule or a single Xe atom. The CO functionalization was achieved by applying a set-point of 8 mV /100 pA with the tip over a CO molecule, followed by turning off the feedback and then ramping the sample bias from zero to 2.6 V. A sudden decrease in the current happened at about 2.2 V indicates a successful functionalization. A subsequent scan over another CO showing sharp central protrusion can confirm the functionalization. After finishing with the CO tip, a bias ramping from zero to 3.6 V with feedback turned off can remove the CO while minimizing the perturbation to the structure of the metal tip apex. A sudden change of current at around 3.2 V often indicates a successful removal of the CO.

A second sequence of AFM images of the same molecule was taken with a Xe functionalized tip. The Xe functionalization was achieved by applying a set-point of 100 mV / 100 pA, followed by turning off the feedback and then bringing the tip into contact with a cluster of Xe atoms. A sudden decrease in current happened at ~3.5 Å advanced from the starting position indicates a successful transfer of a Xe atom. An STM scan with set-point of 100 mV / 100 pA capable of resolving individual Xe atoms inside the Xe cluster can further confirm such a functionalization.

The second experiment with PTCDA molecules (Sigma-Aldrich; purity 97%) was done in a similar manner, except the PTCDA molecules were deposited onto the Cu(111) substrate at about 200 K using thermal sublimation.

The third experiment was done with water molecules (Sigma-Aldrich SKU38796; deionized). The water was purified before deposition, it was firstly boiled at 100°C to rid of any residual gas inside, and was then degassed thoroughly *via* several freeze-pump-thaw cycles. During the experiment, water molecules were deposited *via* a variable leak valve **3** aiming directly at the Cu(111) inside the scanner held at 5 K. The sample was subsequently heated up to

40 K, so that water molecules started to form clusters [7]. The sample was cooled back to 5 K thereafter. Xe and CO were deposited onto the surface with the same procedure as before.

Extended results

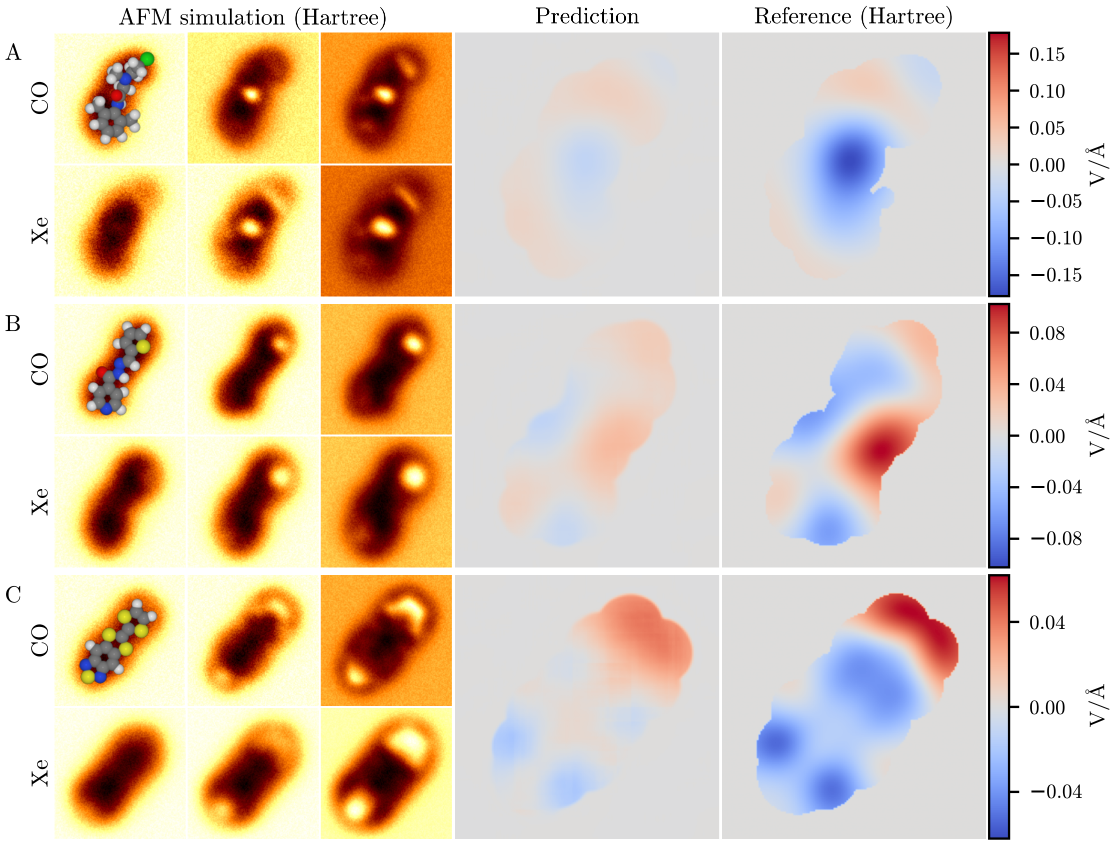

Figure S7: Predictions for the benchmark examples using the DFT Hartree potential for electrostatics in the simulations. Compare to Fig. 2 in the main article.

Single-channel measurements

Since the two-tip measurement presents an additional experimental challenge, we also try training a model using only a single-tip input of CO-AFM. This model is the same as the two-tip model, except that it lacks the other branch of layers in the beginning of the network. Fig. S9A

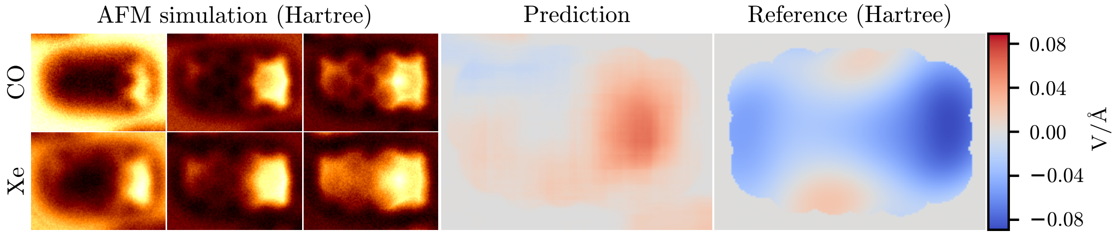

Figure S8: Prediction and reference for on-surface geometry of PTCDA using the DFT Hartree potential for electrostatics in the AFM simulations and for the reference ES Map descriptor.

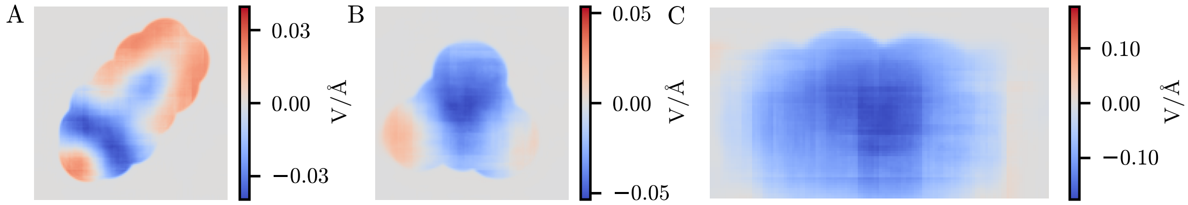

Figure S9: Single-tip characterization of the benchmark examples in the main paper. Predictions are shown for (A) simulated data of TTF-TDZ and experimental data of (B) BCB and (C) PTCDA.

shows an example prediction on simulated data of the TTF-TDZ molecule. At a glance the prediction matches really well with the reference, but a closer inspection reveals that the magnitude of the field is not as accurate. The relative error for the prediction is 5.56%, more than twice the value for the two tip model. When measured on the whole test set, the average loss for the single-tip model is 77% higher than for the two-tip model. Therefore, the single-tip model is less robust, but performance is not fatally worse.

We also apply the single-tip model to experimental data of BCB and PTCDA (Fig. S9B,C) and find that the predictions are not very sensible. For BCB the model predicts mostly negative charge over the whole molecule with some positive regions over two of the halides. This does not match with either of the reference descriptors, where we expect to find the halides to be the most negative regions. The prediction for the PTCDA is similarly biased towards negative values in the middle of the molecule which in both reference descriptors is the least negative

region. These results show that currently the addition of second channel of information is necessary to make the prediction work. Still, the relatively good performance on the simulations indicates that if the simulation model could be improved to be more accurate, then possibly even a single-channel measurement could be used for prediction.

Other tip combinations

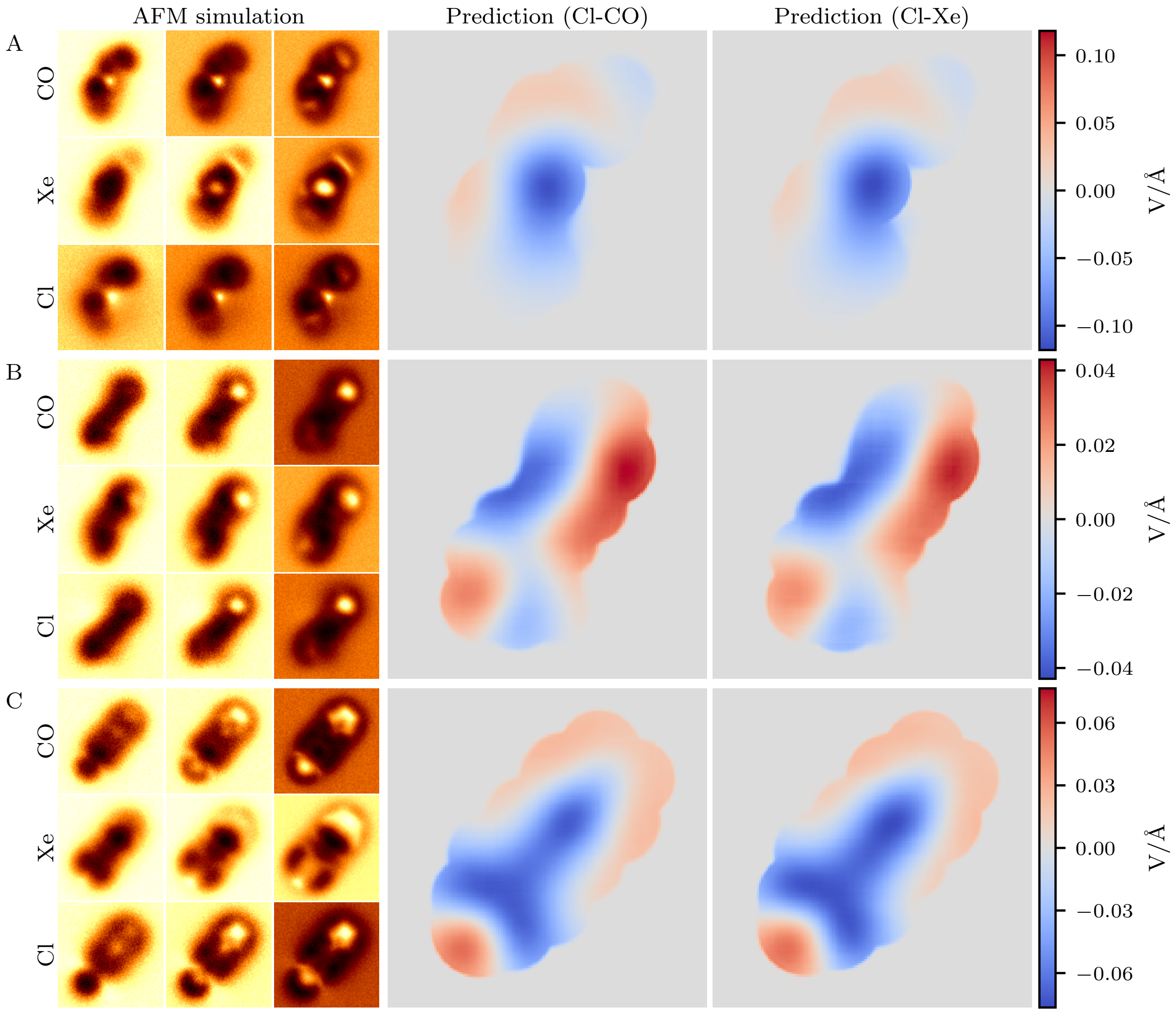

Figure S10: Predictions with models trained on Cl-CO and Cl-Xe tip combinations on the benchmark examples. Compare to Fig. 2 in the main article.

In order to show that the specific combination of CO and Xe tips is not special, we also generate simulations with the Cl tip and train models using the alternative tip combinations of Cl-CO and Cl-Xe. Figure S10 shows example predictions for both tip combinations on simulations of the three benchmark examples introduced in the main article. On these examples, we find that the performance is roughly on par with the CO-Xe model, and for the losses on the test set we even find that the Cl-CO and Cl-Xe models have lower average losses than the CO-Xe model, by 46% and 43%, respectively. In principle, any combination of tips can be used, as long as accurate simulated training data can be generated for them.

Distance dependence

The model is trained to take in AFM image stacks with six constant-height slices for both tips. In training the model, we consciously choose the tip-sample distance to be in range where the furthest images are mostly in the attractive regime, where only the overall shape of the molecule can be distinguished, and the closest images are in the repulsive regime, where at least some sharp atomic features are seen. However, we do not want to go too close to the molecule for two reasons. First, at close range the simulation data used for the training is less representative of the experimental case due to the simulation not taking into account any tip-induced relaxation of the sample. Second, at very close range the interaction between the tip and the sample is dominated by Pauli repulsion and the role of the electrostatics decreases.

In the experiments we have more than six slices for each measurement (see Figs. S6 and S6), which leaves us with some room to choose which subset of images we use for the prediction. This selection process is still not automated and we have to use some judgment in choosing what is the best range for the data so that it best matches the training data, though we do augment the training with a variable range of distance in an attempt to be robust against variations in the distance. In Fig. S11 we explore for two of our experimental cases, BCB and PTCDA, what

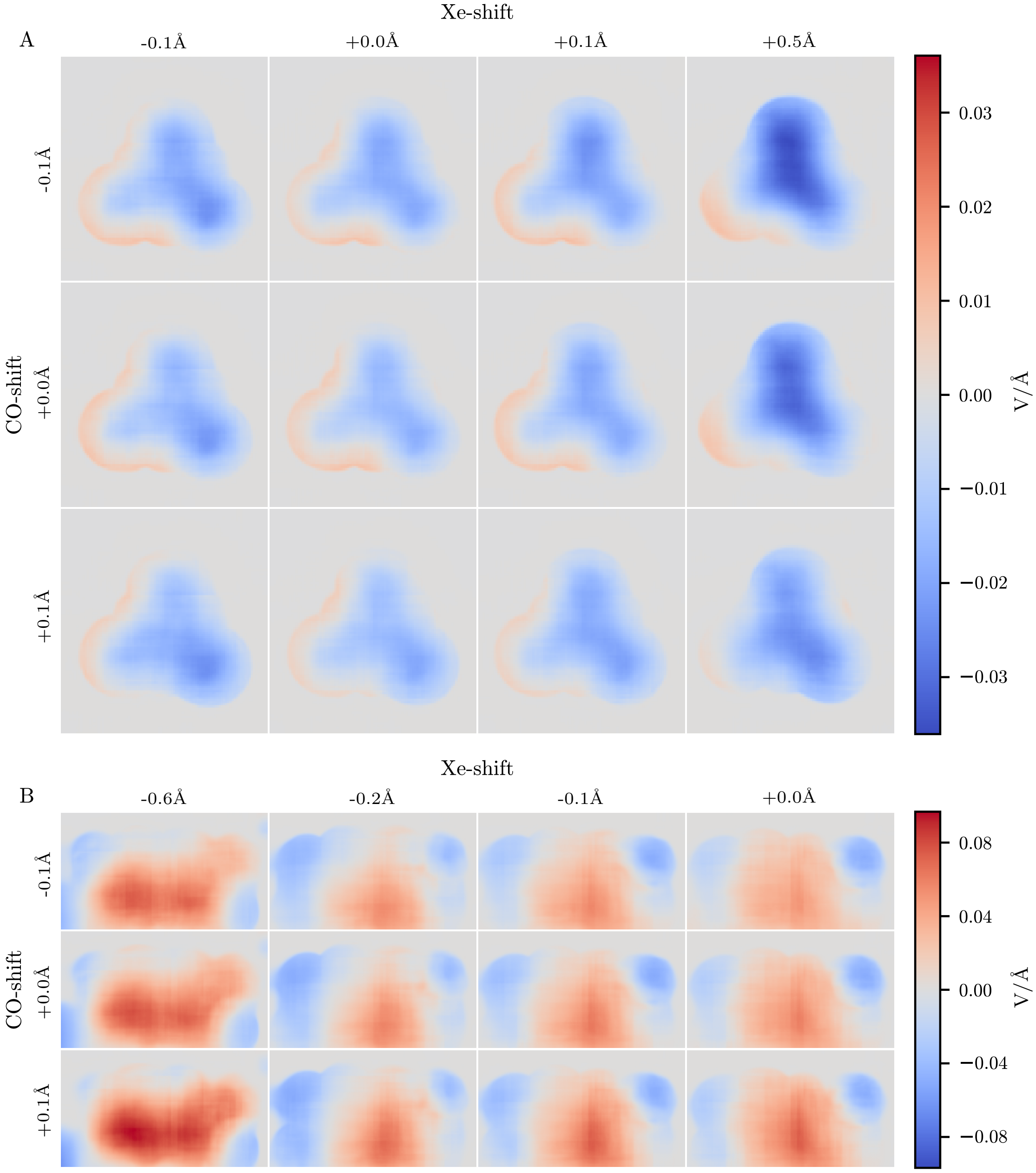

Figure S11: Predictions at different tip-sample distances for experimental images of (A) BCB and (B) PTCDA. On each row the CO-AFM input has been shifted closer or further and on columns correspondingly the Xe-AFM input has been shifted. Here, the shift of +0.0 Å corresponds to the distance used in the predictions in Figs. 3 and 4 of the main article. Negative shift corresponds to smaller tip-sample distance.

happens to the predictions when we vary the tip-sample distance of either the CO or the Xe tip. In both cases we find that small deviations within a range of 0.2 Å do not change the predictions significantly. We also try larger deviations of +0.5 Å for BCB and -0.6 Å for PTCDA of the Xe tip to show that too large deviations start to alter the predictions.

Possible extra electron in PTCDA

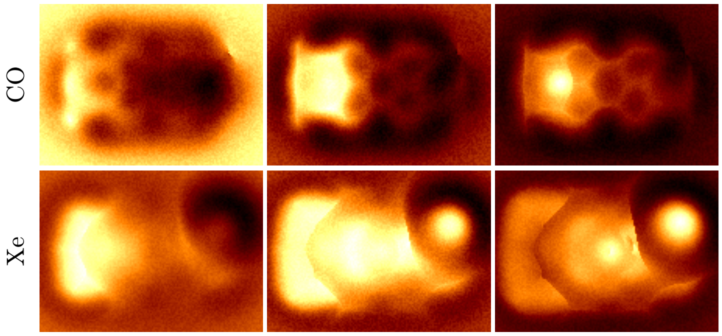

Figure S12: AFM simulations of the on-surface PTCDA with one extra electron added to the top right oxygen. Note that the geometry has been flipped left-to-right compared to Fig. S8A.

In our experimental Xe-AFM images of PTCDA (Fig. S6D) we noted that there appears an unusual bright feature over the oxygen at the top right of the images. We speculate that this feature could be due to an extra electron trapped on that oxygen based on simulations with such an extra electron resulting in a similar bright feature, shown in Fig. S12. In this simulation we took the on-surface geometry used for the DFT Hartree simulation in Fig. S8A and did the simulation using Mulliken charges but adding an extra charge of $-1\,\mathrm{e}$ to the oxygen at the top right of the molecule. The result is a large bright spot surrounded by a dark halo in the Xe-AFM image, somewhat similar to the one in the experimental images. Furthermore, in the CO-AFM simulation there appears a sharp change in contrast over the same oxygen, which on the closer distance makes the oxygen appear very dim compared to the rest of the molecule, which also bears similarity to the experimental images (Fig. S6C). It is, however, unclear if such extra

electron would stay trapped on the oxygen and not transfer to the substrate or tip even when the molecule is being pushed by the AFM probe.

Surface tilt effect on model predictions

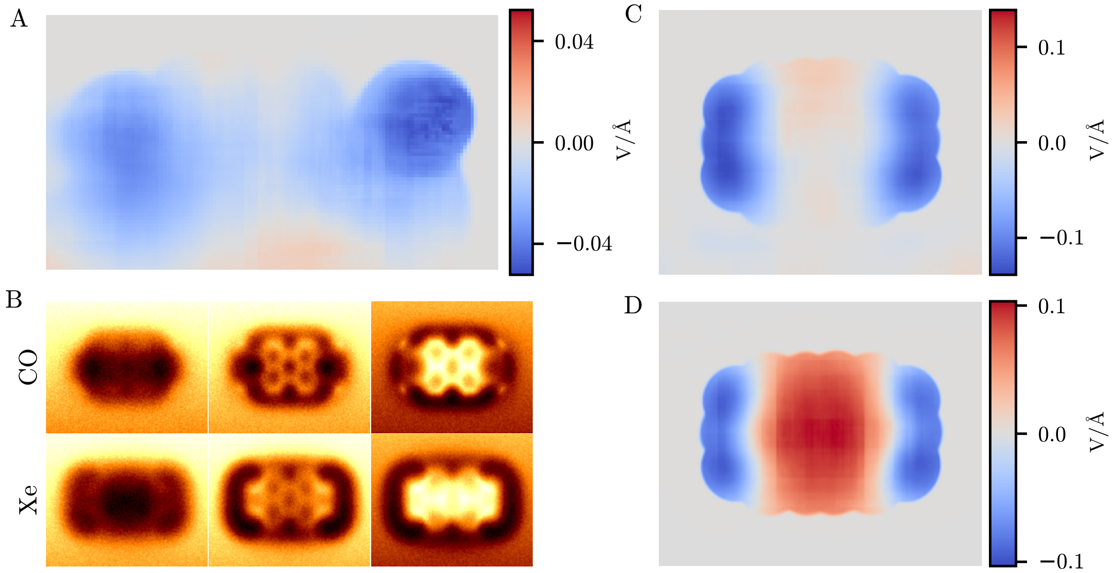

Figure S13: Surface tilt effect on model predictions. (A) ES Map prediction of experimental AFM images of PTCDA on a model trained without background gradient augmentation. (B) Simulated AFM images of PTCDA with added background gradient. Using the input data in (B) we predict the ES Map on models trained (C) without and (D) with the background gradient augmentation.

The PTCDA dataset presented us with the additional challenge that the experiment was performed at a slight tilt which resulted in a gradient in the background of the image. When we artificially added a similar gradient to the simulated AFM images of PTCDA (Fig. S13B), the prediction (Fig. S13C) failed by incorrectly predicting the positive region in the middle of the molecule as being close to neutral. The prediction of the experimental data (Fig. S13A) showed a similar pattern.

Motivated by this finding, we augmented the training of the model with these background gradients, implemented by adding a plane with a set gradient to each AFM image set. The

direction of the gradient is uniform random, and the magnitude of the gradient is randomized such that the range of values in the plane is at most 30% of the range of values in the image set. The zero-point of the plane is always at the center of the images. With this augmentation, we found that the predictions improved both on the simulated images (Fig. S13D) and the experimental prediction also became more consistent. Being robust against these kinds of tilts could be useful in situations where a tilted planar section of a molecule needs to be characterized [8].

References

(1) Ronneberger, O.; Fischer, P.; Brox, T. U-Net: Convolutional Networks for Biomedical Image Segmentation 2015, 1505.04597. arXiv, `http://arxiv.org/abs/1505.04597` (accessed 11/06/2021).

(2) Oktay, O.; Schlemper, J.; Folgoc, L. L.; Lee, M.; Heinrich, M.; Misawa, K.; Mori, K.; McDonagh, S.; Hammerla, N. Y.; Kainz, B.; Glocker, B.; Rueckert, D. Attention U-Net: Learning Where to Look for the Pancreas 2020, 1804.03999. arXiv, `http://arxiv.org/abs/1804.03999` (accessed 11/06/2021).

(3) Xu, B.; Wang, N.; Chen, T.; Li, M. Empirical Evaluation of Rectified Activations in Convolutional Network 2015, 1505.00853. arXiv, `http://arxiv.org/abs/1505.00853` (accessed 11/06/2021).

(4) Kingma, D. P.; Ba, J. Adam: A Method for Stochastic Optimization 2017, 1412.6980. arXiv, `http://arxiv.org/abs/1412.6980` (accessed 11/06/2021).

(5) Alldritt, B.; Hapala, P.; Oinonen, N.; Urtev, F.; Krejci, O.; Canova, F. F.; Kannala, J.; Schulz, F.; Liljeroth, P.; Foster, A. S. Automated Structure Discovery in Atomic Force Microscopy. *Sci. Adv.* **2020**, *6*, eaay6913.

(6) Hapala, P.; Kichin, G.; Wagner, C.; Tautz, F. S.; Temirov, R.; Jelínek, P. Mechanism of High-Resolution STM/AFM Imaging with Functionalized Tips. *Phys. Rev. B* **2014**, *90*, 085421.

(7) Liriano, M. L.; Gattinoni, C.; Lewis, E. A.; Murphy, C. J.; Sykes, E. C. H.; Michaelides, A. Water–Ice Analogues of Polycyclic Aromatic Hydrocarbons: Water Nanoclusters on Cu(111). *J. Am. Chem. Soc.* **2017**, *139*, 6403–6410.

(8) Albrecht, F.; Pavliček, N.; Herranz-Lancho, C.; Ruben, M.; Repp, J. Characterization of a Surface Reaction by Means of Atomic Force Microscopy. *J. Am. Chem. Soc.* **2015**, *137*, 7424–7428.
